# Discovery of bimodal hepatitis B virus ribonuclease H and capsid assembly inhibitors

**DOI:** 10.1371/journal.ppat.1012920

**Published:** 2025-02-10

**Authors:** Daniel P. Bradley, Caleb J. Valkner, Qilan Li, Makafui Gasonoo, Marvin J. Meyers, Georgia-Myrto Prifti, Dimitrios Moianos, Grigoris Zoidis, Adam Zlotnick, John E. Tavis

**Affiliations:** 1 Department of Molecular Microbiology and Immunology, Saint Louis University School of Medicine, Saint Louis, Missouri, United States of America; 2 Saint Louis University Institute for Drug and Biotherapeutic Innovation, Saint Louis, Missouri, United States of America; 3 Department of Molecular & Cellular Biology, Indiana University, Bloomington, Indiana, United States of America; 4 Department of Chemistry, Saint Louis University, Saint Louis, Missouri United States of America; 5 Department of Pharmacy, Division of Pharmaceutical Chemistry, School of Health Sciences, National and Kapodistrian University of Athens, Panepistimiopolis Zografou, Athens, Greece; Pennsylvania State University College of Medicine: Penn State College of Medicine, UNITED STATES OF AMERICA

## Abstract

Hepatitis B virus (HBV) ribonuclease H (RNaseH) inhibitors are a potent class of antivirals that prevent degradation of the viral pregenomic RNA during reverse transcription and block formation of mature HBV DNAs. Development of HBV RNaseH inhibitors is entering advanced preclinical analyses. To ensure the mechanism of action was fully understood, we defined the effects of RNaseH inhibitors on other steps of HBV replication. Some *N*-hydroxypyridinedione (HPD) HBV RNaseH inhibitors significantly reduced accumulation of capsids in HBV-replicating cells. A representative HPD **1466**, with a 50% effective concentration against HBV replication of 0.25 µM, decreased capsid and core protein accumulation by 50–90% in HepDES19 and HepG2.2.15 cells. Surprisingly**, 1466** did not affect pregenomic RNA encapsidation, demonstrating a specific effect on empty capsids. HBV genomic replication was not necessary for **1466**’s inhibitory effect as it decreased capsid accumulation in cells transfected with replication-deficient mutants blocking pgRNA encapsidation (Δ-bulge), DNA synthesis (YMHA), and RNaseH (D702A) activities. **1466** also decreased capsid and core protein accumulation in cells transfected with a core protein expression plasmid, indicating that other HBV products are unneeded. **1466** reduced initial capsid assembly rates in biochemical assembly reactions employing purified core protein (Cp149), demonstrating a specific effect on HBV core protein. We conclude that the bimodal HPD HBV RNaseH inhibitor **1466** is the prototypic member of a new class of capsid assembly modulators (CAM) that inhibits capsid assembly rather than accelerating it, as all other CAM classes do. We propose that this class be called CAM-I, for *CAM-inhibitor*. These results lay the foundation for identifying bimodal HBV antivirals targeting the RNaseH and capsid assembly.

## Author summary

Hepatitis B virus (HBV) is a leading cause of liver cirrhosis, liver failure, and hepatocellular carcinoma worldwide. Current treatments reduce but do not eliminate disease progression, and they do not cure chronic HBV infections. We are pursuing the HBV ribonuclease H (RNase H) as a new drug target and identified *N*-hydroxypyridinediones (HPDs) as promising RNase H inhibitors. While exploring the effects of HPDs on cells, we found some of them had an unexpected second function of specifically eliminating empty HBV capsids without affecting levels of nucleocapsids that carry the viral genome. This secondary activity qualifies these HPDs as capsid assembly modulators (CAM), a promising class of drugs under development against HBV. The HPDs reduce capsid accumulation in part by slowing the rate of capsid assembly rather than accelerating it as do all other CAMs. This defines the HPDs as a new class of CAMs that we propose be called CAM-I, for CAM-inhibitors. The dual activity of CAM-I compounds may help promote HBV clearance in patients, particularly if the CAM function can be optimized to also target nucleocapsids that are the precursors to infection HBV virions.

## Introduction

Hepatitis B virus (HBV) is a hepatotropic partially double-stranded DNA virus that chronically infects an estimated 254 million people worldwide and causes ~1 million deaths annually [1,2]. Current antiviral therapies primarily employ nucleos(t)ide analogs (NA) which block viral DNA synthesis and drive HBV titers to near or below clinical detection levels but are not curative [3,4]. Therefore, treatment is usually lifelong. The development of new anti-HBV therapies has focused on targeting multiple aspects of the viral replication cycle that will work in concert with current therapeutics to identify a functional cure that maintains undetectable viral levels after treatment termination [5].

HBV replicates by protein-primed reverse transcription within viral nucleocapsids. The HBV polymerase protein (P) replicates the viral genome and has two enzymatically active domains essential for viral replication, the reverse transcriptase (RT) and ribonuclease H (RNaseH) [6]. The RNaseH degrades the viral pregenomic RNA (pgRNA) as the RT copies it to make the first DNA strand. We have established the HBV RNaseH as an effective target for inhibiting HBV replication and developed a screening pipeline for identifying RNaseH inhibitors [7–10]. The leading chemotypes of RNaseH inhibitors include the α-hydroxytropolones (αHT), *N*-hydroxynapthyridinones (HNO), and *N*-hydroxypyridinediones (HPD) [11] that act by coordinating the two Mg^++^ ions in the active site of the HBV RNaseH. Inhibiting the RNaseH produces RNA:DNA heteroduplexes within nucleocapsids and prevents formation of the mature relaxed circular DNA (rcDNA) genome [7]. Development of potent HBV RNaseH inhibitors is ongoing, and drugs targeting the RNaseH are expected to be used in combination with other antiviral therapies to augment inhibition of HBV replication [12].

As part of our development of HBV RNaseH inhibitors, we sought to define their effects on each step of HBV replication, beginning with capsid accumulation. We hypothesized that HBV-RNaseH inhibitors could destabilize the HBV nucleocapsid due to the metastable nature of the capsid and the increased rigidity of RNA:DNA heteroduplexes compared to single-stranded RNA, DNA, and double-stranded DNA [13,14], resulting in capsid disruption and degradation.

The HBV capsid is an icosahedral structure composed of 120 homodimers of the HBV core protein (HBc) [15,16]; nucleocapsids are capsids containing HBV nucleic acids. Capsid assembly modulators (CAMs) are HBV replication inhibitors that accelerate capsid assembly and prevent pgRNA encapsidation [15,17]. All known CAMs bind to the same hydrophobic pocket at the HBc dimer-dimer interface, stabilizing the interaction [17–19]. There are two functionally defined classes of CAMs: CAM-Es, which induce formation of genome-free (*empty*) capsids, and CAM-As, which induce formation of large non-icosahedral *aberrant* capsids [17,18]. CAM-Es increase empty capsid accumulation while decreasing nucleocapsid levels, whereas CAM-As, by favoring aberrant assembly, decrease capsid and nucleocapsid accumulation and can promote autophagy [20] and apoptosis of HBV-replicating hepatocytes [21].

Here we describe a chemotype of HBV RNaseH inhibitors that inhibit the rate of capsid assembly and reduce capsid accumulation. These studies reveal a new class of bimodal HBV RNaseH- and HBc-targeting inhibitors with distinct effects on capsids compared to other CAMs.

## Results

### RNaseH inhibitors can alter capsid accumulation

**Fig 1:**
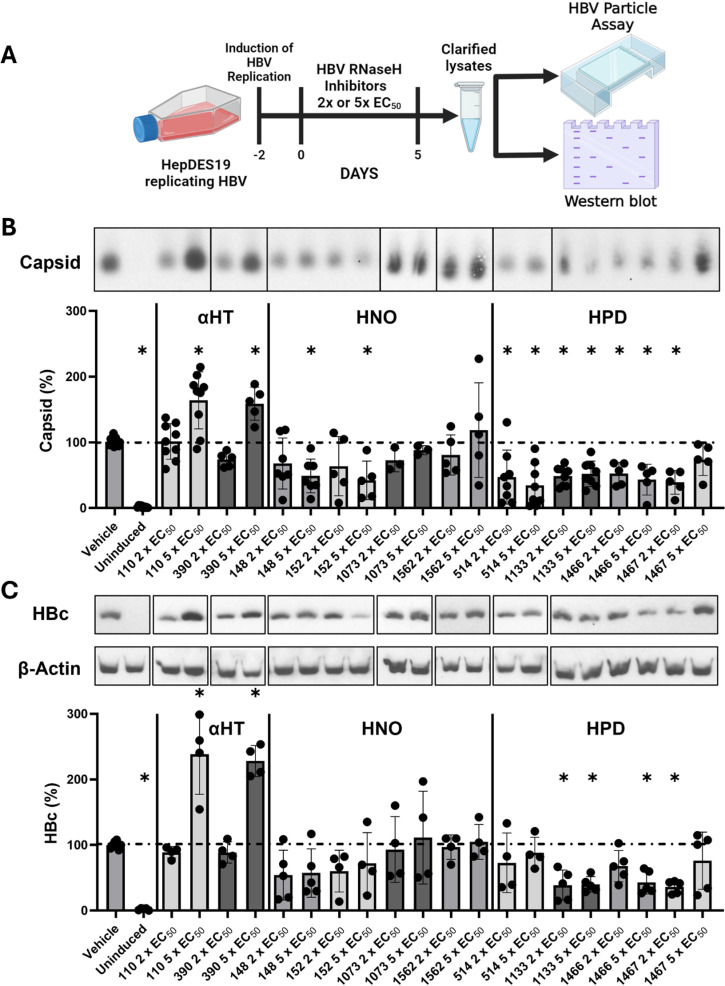
Screening HBV RNaseH inhibitors in HepDES19 cell for effects on capsids and core protein. HepDES19 were treated with 2x or 5x EC_50_ of the indicated HBV RNaseH inhibitors for five days. Protein-balanced cytoplasmic lysates were resolved by native agarose gel electrophoresis or denaturing SDS-PAGE (**A**). Capsid accumulation and HBc protein levels were measured by the HBV particle assay (**B**) or western blot (**C**). Capsid and HBc levels were measured by densitometry and normalized to the vehicle control. One-way ANOVA with Dunnett’s multiple comparisons test between vehicle and compound treated conditions determined significance. * P < 0.05. Panel **A** was made with Biorender.com.

HBV RNaseH inhibitors block viral replication and produce large RNA:DNA heteroduplexes [7] that are stiffer than double-stranded DNA (dsDNA) [13,14]. Due to the capsid’s small size and metastable nature [22–24], we hypothesized that the RNA:DNA heteroduplex could exert a greater force than dsDNA on the nucleocapsid, potentially disrupting them. Therefore, we screened ten HBV RNaseH inhibitors from three chemotypes at 2x or 5x their 50% effective concentrations (EC_50_) against HBV replication for effects on capsid accumulation in replicating HepDES19 cells, a line carrying a tetracycline-repressible HBV pgRNA expression cassette [25]. Compound structure and representative replication inhibition assays demonstrating preferential suppression of the HBV plus-polarity DNA strand that is a characteristic of RNaseH inhibition are shown in S1 Fig. Capsid accumulation was assessed in protein-normalized cytoplasmic lysates after five days of compound exposure by the HBV particle assay [26], and HBc protein levels were assessed in the same samples by western blot (Fig 1).

The αHT compounds **110** and **390** significantly increased capsid and HBc protein levels relative to the vehicle control at 5x EC_50_. Two of the HNOs (**148** and **152**) significantly decreased capsid accumulation at 5x EC_50_, while the others (**1073** and **1562**) did not. None of the HNOs significantly affected HBc levels. Conversely, all HPDs (**514**, **1133**, **1466**, and **1467**) significantly decreased capsid accumulation while modestly decreasing HBc levels. A follow-up experiment identified two additional HPD compounds (**1736** and **1740**) that suppressed capsid accumulation by >50% at 10 µM in HepDES19 cells replicating HBV (S2 Fig). Therefore, HBV RNaseH inhibitors affected capsid accumulation in a compound- and chemotype-dependent manner and affected HBc levels to a lesser degree.

### HBV RNaseH inhibitors affect capsid accumulation in the absence of HBV DNA synthesis

**Fig 2:**
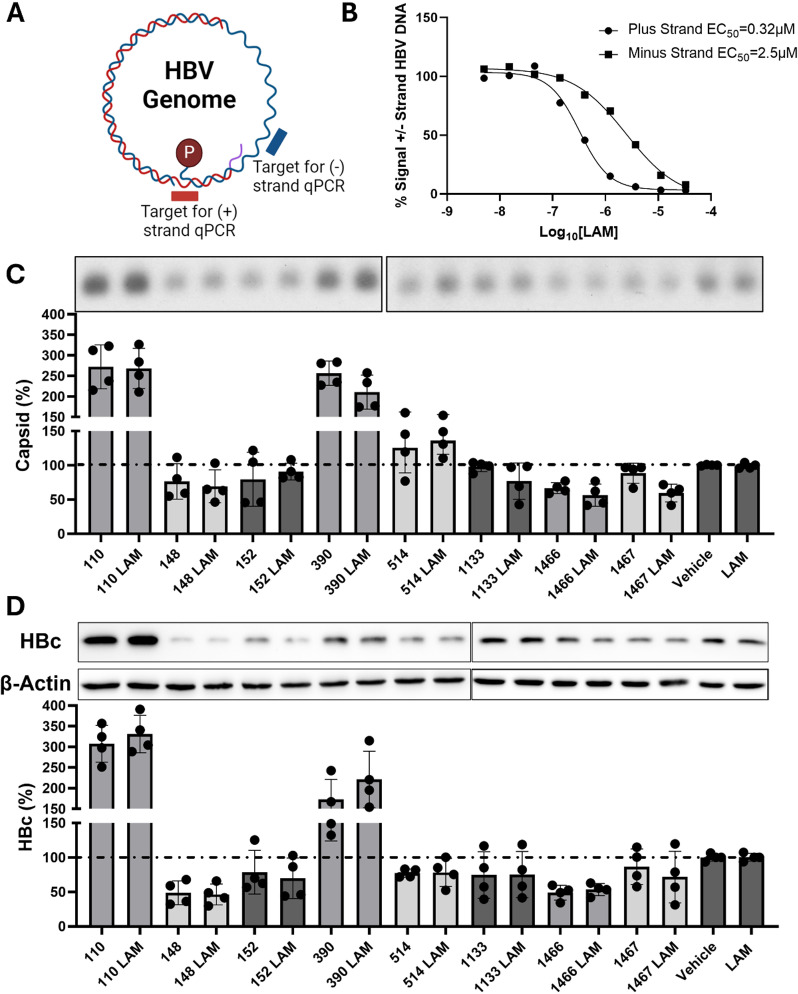
HBV RNaseH inhibitors effects on capsid accumulation occur independent of HBV DNA synthesis. HBV genome map showing the location of primers used for strand-preferential qPCR replication inhibition assays (**A**). Quantification of both plus- and minus-polarity DNA strands and dose-response curve of **LAM** treatment in an HBV replication inhibition assay (**B**). HepDES19 cells were treated with 5x EC_50_ of the indicated HBV RNaseH inhibitor with 20 μM of **LAM**. Intracellular HBV capsid accumulation was detected with HBV particle assays (**C**), and intracellular HBc protein levels were detected by western blot (**D**). Data were normalized to vehicle-treated samples, and a one-way ANOVA with a Sidek comparison between RNaseH inhibitor treatment and LAM with RNaseH inhibitor treatment (e.g., **110** and **110** **LAM**) was performed. All comparisons were non-significant (P > 0.05). Panel **A** was made with Biorender.com.

We presumed that the HBV RNaseH inhibitors’ effects on capsid accumulation were dependent on inhibition of the RNaseH. Therefore, we tested whether HBV DNA synthesis and its associated RNaseH activity were necessary for the inhibitors’ effects on capsid accumulation. The nucleoside analog Lamivudine (**LAM**) is an approved HBV antiviral drug that can inhibit all detectable HBV DNA synthesis at low micromolar concentrations in the HBV strand-preferential qPCR replication inhibition assay (Fig 2A and 2B) [27]. We used 20 μM of **LAM** to block HBV DNA synthesis and preclude the HBV RNaseH activity by preventing formation of RNA:DNA heteroduplexes that are the substrate for the RNaseH. We hypothesized that **LAM** co-treatment would ameliorate the HBV-RNaseH inhibitors’ effects on capsid accumulation.

HepDES19 cells replicating HBV were treated with 5x EC_50_ of eight compounds that have varying effects on capsid accumulation with or without 20 μM of **LAM** for five days. Capsid and HBc levels were detected by the HBV particle assay and western blot, and values were normalized to the corresponding vehicle or **LAM** treatment control. **LAM** treatment did not alter the compound-mediated effects on capsid accumulation or HBc levels induced by αHTs **110** and **390**, HNO **148,** and HPD **1466** (Fig 2C and 2D). These results indicate that HBV genomic replication, formation of RNA:DNA heteroduplexes, and RNaseH activity are not necessary for these HBV-RNaseH inhibitors to alter capsid accumulation. An additional experiment using 10 μM of the nucleoside analog Entecavir instead of **LAM** yielded a similar result. Collectively, these data refute the hypothesis that capsid suppression would be dependent on RNaseH inhibition.

Figs 1 and 2 demonstrate that some HBV RNaseH inhibitors significantly affect HBV capsid accumulation; however, the disparate effects on capsid accumulation during treatment with αHTs and HPDs suggest that they affect capsid accumulation by different mechanisms. The αHT **390** and HPD **1466** were selected as representative inhibitors that increased and decreased capsid accumulation during subsequent mechanistic analyses.

### αHT 390 increases HBV capsid accumulation by a reactive oxygen species-dependent mechanism

**Fig 3:**
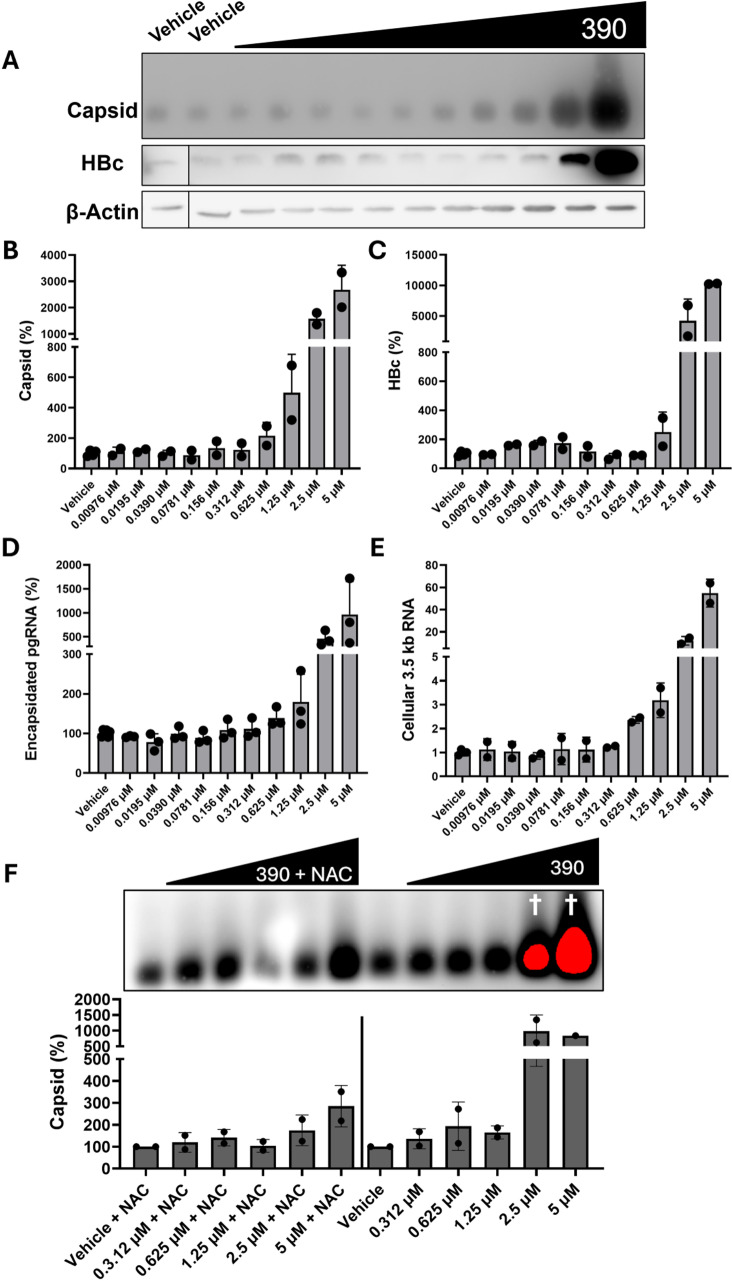
Compound 390 induces HBV capsid accumulation via an ROS-mediated mechanism. HepDES19 cells were treated with the indicated concentrations of **390** for four days with 20 µM **LAM** cotreatment. Capsids were detected by HBV particle assay, HBc protein was detected by western blot, and β-actin was measured by western blot as a loading control. (**A**). Quantification of HBV capsid (**B**), HBc (**C**), encapsidated pgRNA measured by qPCR of RNA from isolated capsids (**D**), and cellular 3.5 kb HBV RNA measured by qPCR of RNA from whole-cell lysates (**E**). Capsid accumulation from HepDES19 cells treated with a titration of **390** with or without 10 mM of the antioxidant **NAC** for four days (**F**). Samples measured from saturated signals are indicated with †. All data were normalized to vehicle-treated samples from the same experiment. N=1 for the 5 μM treatment in panel **F** due to cytotoxicity.

αHTs are a well-studied chemotype of HBV RNaseH inhibitors with *in vitro* and *in vivo* efficacy [28–30]. Recently, we determined that **110**, **390**, and related tropolones induce cytotoxicity by a reactive oxygen species (ROS)-mediated mechanism [31]. **390** is a potent inhibitor of HBV replication with an EC_50_ of 0.31 µM [28], but it paradoxically increased capsid accumulation in HepDES19 cells replicating HBV (Figs 1 and 2). To evaluate **390**’s effect on HBV replication we measured HBV capsid, HBc, encapsidated pgRNA, and cytoplasmic HBV 3.5 kb RNA levels in HepDES19 cells during **390** and **LAM** co-treatment. **390** increased all measures of HBV replication by ten-fold at micromolar concentrations (Fig 3C to 3E), indicating strong induction of HBV gene expression and/or robust reduction in degradation of the HBV products. However, **390** is cytotoxic at similar micromolar concentrations [29,31], and cytotoxicity was noted in this assay. We hypothesized that **390** induced HBV gene expression and capsid accumulation by a cytotoxicity-dependent mechanism. N-acetylcysteine (NAC) can ameliorate tropolone-induced ROS production and cytotoxicity [31]. Therefore, we tested if NAC treatment could decrease **390**’s effect on capsid accumulation. HepDES19 cells were treated with **390** with or without 10 mM of NAC. NAC inhibited **390**’s induction of capsid accumulation by 4- to 5-fold (Fig 3F), indicating that **390** elevated capsid accumulation in large part by inducing ROS/cytotoxicity in HepDES19 cells in which HBV pgRNA transcription is driven by the CMV promoter. In contrast, **390** had no effects on capsid accumulation or encapsidated HBV RNA levels in HepG2.2.15 cells in which HBV transcription is driven from its native promoters (S3 Fig). ROS-mediated NFκB signaling can induce gene expression from the immediate early CMV promotor [32–34] which controls HBV pgRNA expression in HepDES19 cells. We concluded that tropolones increased capsid accumulation in a cytotoxicity/ROS-mediated mechanism, which was likely an artifact of the CMV promoter-driven HBV expression system. Consequently, analysis of tropolones’ effect on capsid accumulation was halted.

### HPD 1466 inhibits capsid accumulation independently of HBV replication

**1466** is an HBV RNaseH inhibitor with an EC_50_ of 0.25 μM that consistently decreased capsid and HBc accumulation. While the concentration of **LAM** employed in Fig 2 inhibits all detectable HBV DNA synthesis, we sought to confirm that **1466** inhibits capsid accumulation independent of viral DNA replication. Capsid accumulation was measured in cells transfected with a genotype A HBV pgRNA expression plasmid carrying D702A (RNaseH deficient) [35], 5’ Δ-Bulge (pgRNA packaging deficient) [36], or YMHA (RT deficient) [37] mutations. Transfected cells were treated with 10 µM of **1466**, 1 µM of **GLS4**, or a vehicle control. **GLS4** is a CAM-A that induces formation of aberrant HBc aggregates and reduces capsid accumulation [38].

**Fig 4:**
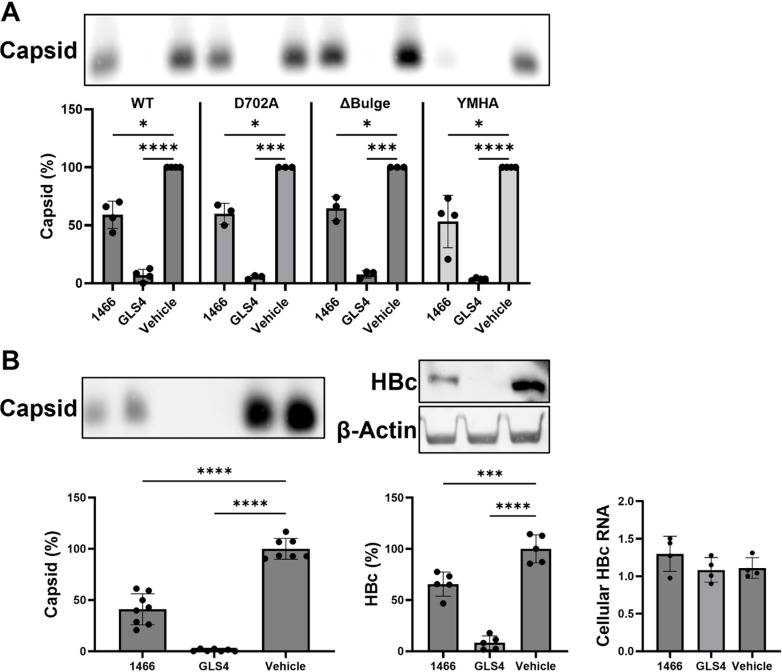
1466 inhibits capsid accumulation independent of HBV replication. HepG2 cells were transfected with the indicated HBV pgRNA expression plasmids (**A**) or core protein expression plasmid (**B**) and treated with 10 µM of **1466** or 1 µM of **GLS4** for two days. Capsid accumulation was measured by the HBV particle assay (**A** and **B**). HBc protein and HBc ORF-containing RNA levels were measured in western blot or RTqPCR (**B**). All data were normalized to vehicle-treated samples from the same experiments, and significance was determined by one-way ANOVA with Dunnett’s multiple comparisons test between vehicle and compound-treated conditions. * P<0.05, ** P<0.01, *** P<0.001, **** P<0.0001.

**1466** significantly decreased capsid accumulation in cells transfected with the WT and all three mutant HBV constructs (Fig 4A), confirming that DNA synthesis and RNA:DNA heteroduplex formation are not necessary for **1466**’s inhibition of capsid accumulation. Furthermore, the RNaseH-deficient mutant is expected to reduce the binding affinity of RNaseH inhibitors because the D702A mutation eliminates a key moiety holding the Mg^++^ ions in the active site and much of the binding energy for the HPDs stems from coordinating the Mg^++^ ions. Consequently, these data also strongly imply that **1466** decreases capsid accumulation without binding to the RNaseH active site.

HBc is the only viral product required to form capsids. Therefore, we tested if **1466** inhibited capsid accumulation in cells expressing just HBc. HepG2 cells were transfected with an HBc expression plasmid (pcDNA3.1-HBc) and treated with 10 μM **1466**, 1 μM **GLS4**, or vehicle control for two days, and then capsid accumulation, HBc, and HBc mRNA levels were measured. As expected, **GLS4** significantly decreased capsid and HBc accumulation but did not affect HBc transcript levels (Fig 4A and 4B). Similarly, **1466** significantly decreased capsid and HBc levels without affecting HBc mRNA levels (Fig 4B). These data demonstrate that **1466** either acts directly on HBc and/or capsids or interacts with cellular factors involved in capsid formation, decreasing capsid and HBc levels in cells.

### HPD 1466 inhibits HBV capsid accumulation without altering levels of encapsidated pgRNA

A titration of **1466** with **LAM** cotreatment was performed to evaluate if **1466** decreases capsid accumulation in a dose-dependent manner. **LAM** co-treatment allows the measurement of encapsidated pgRNA which reflects nucleocapsid accumulation without loss due to its conversion to DNA. Capsid, HBc, whole-cell HBV 3.5 kb mRNA, and encapsidated pgRNA levels were measured after four days of inhibitor treatment in HepDES19, and HepG2.2.15 cells [39]. HepDES19 cells were induced to replicate HBV simultaneously with compound addition, mimicking capsid accumulation patterns in a *de novo* infection. In contrast, HepG2.2.15 cells constitutively express HBV from integrated HBV sequences using the HBV promoters and were treated while capsid levels were at steady-state, mimicking the capsid accumulation pattern in an established infection.

**Fig 5:**
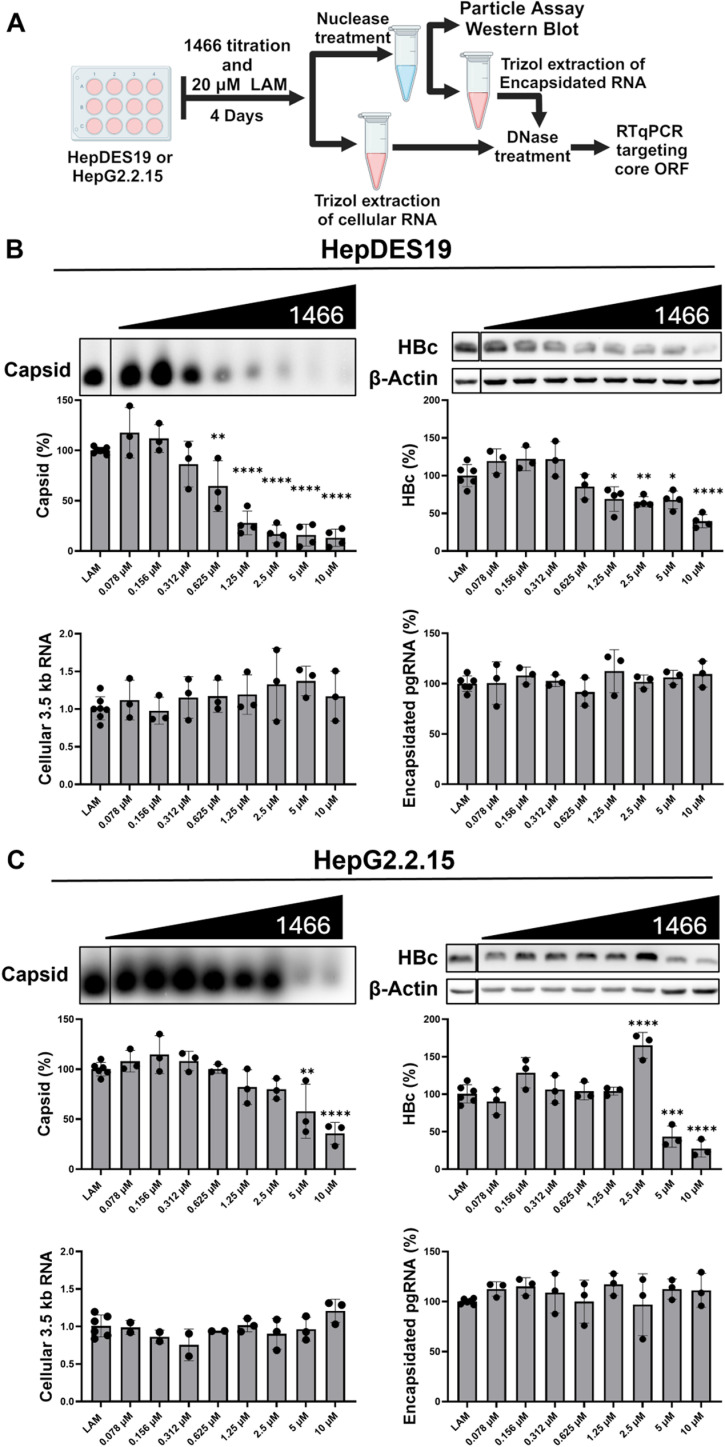
HPD 1466 decreases empty capsid accumulation in cells replicating HBV. Experimental layout (**A**). HepDES19 (**B**) or HepG2.2.15 (**C**) cells were cotreated with **LAM** and a titration of **1466**. Cytoplasmic lysates were treated with nuclease to destroy cytoplasmic DNA and RNA, and then capsid, HBc, and encapsidated pgRNA levels were measured from the same sample and the data were normalized to vehicle-treated samples from the same experiment. Cellular 3.5 kb HBV RNA was measured by RTqPCR and normalized to cellular GAPDH RNA and vehicle-treated samples from the same experiment. Significance was determined by one-way ANOVA with a Dunnett’s multiple comparisons test between vehicle and compound treated conditions. * P<0.05, ** P<0.01, *** P<0.001, **** P<0.0001. Panel **A** was made with Biorender.com.

**1466** decreased capsid accumulation in a dose-dependent manner in HepDES19 and HepG2.2.15 cells by >80% and >50% at 10 µM (Fig 5), with EC_50_ values of 0.54 μM and 4.9 μM, respectively, for capsid suppression. In both cell types, **1466** significantly decreased HBc levels in tandem with capsid suppression without affecting cytoplasmic HBV 3.5 kb transcript levels that encode HBc (Fig 5). This further demonstrates that **1466** is a potent inhibitor of capsid accumulation that acts at the protein level rather than at the transcript level. Surprisingly, **1466** did not affect nuclease-resistant 3.5 kb RNA levels (i.e., encapsidated pgRNA), indicating that **1466** inhibits accumulation of empty capsids but has little effect on nucleocapsids. **1466** inhibited capsid accumulation during both its initial expression (HepDES19) and also from steady-state conditions (HepG2.2.15), but there was nearly ten-fold difference in EC_50_ values between the cell types. This implies that **1466**’s effects on capsid assembly that dominate in HepDES19 cells are substantially stronger than effects on capsid turnover which are most evident in Hep2.2.15 cells in which capsid levels are at steady state upon compound addition.

**1466**’s inhibition of empty capsid accumulation could be a result of increased secretion from the cells, inhibiting capsid assembly, and/or inducing capsid disassembly. Increased secretion of virions is excluded because the effect is seen in HepDES19 cells which cannot make the viral surface glycoproteins and hence cannot form virions. To test if the decline in intracellular capsids was due to enhanced release of naked capsids that is not dependent on the surface proteins, we treated HepDES19 cells replicating HBV with **1466**, collected the cell supernatant over seven days, concentrated the medium, and measured capsid levels in the medium. There was no increase in capsids in medium from **1466**-treated cells compared to vehicle-treated cells (S4A Fig), precluding enhanced release of empty capsids. To test if **1466** was sufficient to disassemble capsids, cytoplasmic lysates from HepDES19 cells replicating HBV were treated with 10 µM of **1466, GLS4** (CAM-A), or **NVR3-778** (CAM-E) for 24 hours and analyzed by the HBV particle assay. **1466** and the two CAMs did not affect capsid levels (S4B Fig), indicating that **1466** is not sufficient to induce disassembly of pre-formed capsids in metabolically inactive cytoplasmic lysates.

### Direct comparison of 1466 to representative CAM-A and CAM-E inhibitors

**Fig 6:**
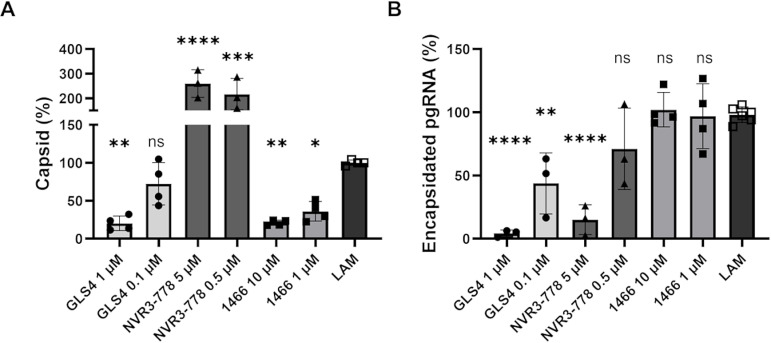
Effects of 1466 on capsid and pgRNA encapsidation compared to known CAM-A and CAM-E inhibitors. Capsid accumulation (**A**) and pgRNA encapsidation (**B**) were measured from HepDES19 cells treated with the indicated inhibitor for four days. Data were normalized to **LAM**-treated samples from the same experiment. Significance was determined by one-way ANOVA with Dunnett’s multiple comparisons test between vehicle and compound-treated conditions. * P<0.05, ** P<0.01, *** P<0.001, **** P<0.0001.

We next directly compared **1466** to other known HBV capsid-targeting antivirals due to its unusual ability to suppress accumulation of empty capsids without affecting levels of pgRNA-containing nucleocapsids. HepDES19 cells were treated with **LAM** plus **GLS4** (CAM-A), **NVR3-778** (CAM-E), or **1466**. Capsids and encapsidated pgRNA were measured by the HBV particle and encapsidation assays (Fig 6). **GLS4** reduced capsid accumulation, **NVR3-778** increased capsid accumulation, and both decreased pgRNA encapsidation, consistent with their established mechanisms of action. In contrast, **1466** decreased capsid accumulation (Fig 6A) without affecting encapsidated pgRNA (Fig 6B). **1466**’s effects were therefore distinct from the patterns that define the CAM-A and CAM-E mechanisms, demonstrating that it inhibits capsid accumulation by a previously undescribed mechanism that preferentially targets empty capsids over nucleocapsids.

### 1466 inhibits capsid formation in a biochemical assay with purified HBc

Finally, we tested whether the HPD **1466** directly affects capsid assembly by screening it in a well-established biochemical capsid assembly assay [40] which uses purified Cp149, a C-terminal truncated HBc that auto-assembles into capsids upon increasing NaCl concentration. Capsid assembly was measured by light scattering to reveal the relative assembly rate and yield of capsids [40,41]. All known CAMs substantially increase both the capsid assembly rate and yield in this assay. Furthermore, capsid morphologies formed in the presence of a compound can be used to describe the class of CAM [15,17].

**Fig 7:**
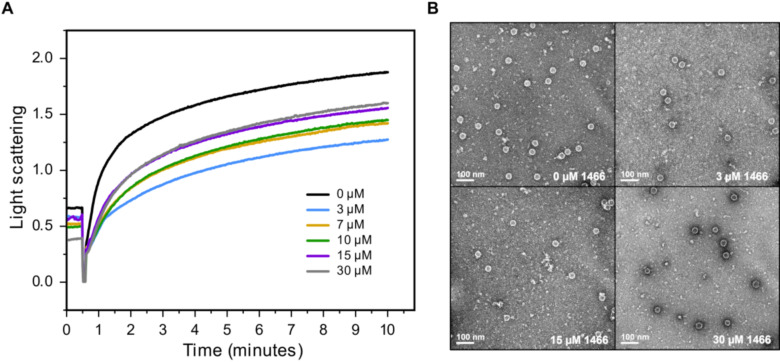
1466 slows the capsid assembly rate in biochemical Cp149 capsid assembly reactions. The C-terminally truncated HBc derivative Cp149 was purified and induced to assemble in biochemical reactions by addition of NaCl. Capsid assembly rate curves in the presence of varying concentrations of **1466** were acquired by measuring light scattering (**A**). Cp149 was treated with the indicated concentration of **1466** prior to initiation of capsid assembly with 300 mM NaCl at 30 seconds. Capsids assembled in the presence of **1466** in panel **A** were stained and visualized by electron microscopy (**B**).

Cp149 was mixed with a titration of **1466**, and capsid assembly was initiated by addition of 300 mM NaCl. Fig 7A shows a representative assay reporting the average of three technical replicates for each compound concentration. In the presence of **1466**, the rate of assembly was slower than in the vehicle-treated control reaction for all compound concentrations. Capsids formed in the presence of **1466** had normal T = 4 morphologies in electron micrographs (Fig 7B). In the absence of aberrant assembly, capsid assembly reactions contain almost exclusively dimers and capsids, with intermediates making an inconsequential contribution [42]. Thus, the change in light scattering shows that the forward rate of assembly is slower by about a factor of two (compare the relative light scattering at 3–5 minutes). The reactions containing **1466** yielded the same amount of capsid as those lacking the compound when the reactions were allowed to approach equilibrium (24 hours) (S5 Fig). These data show that **1466** reduces the rate of capsid assembly from purified HBc (Cp149), providing a mechanistic rationale for the decreased capsid accumulation in cells.

## Discussion

**Fig 8:**
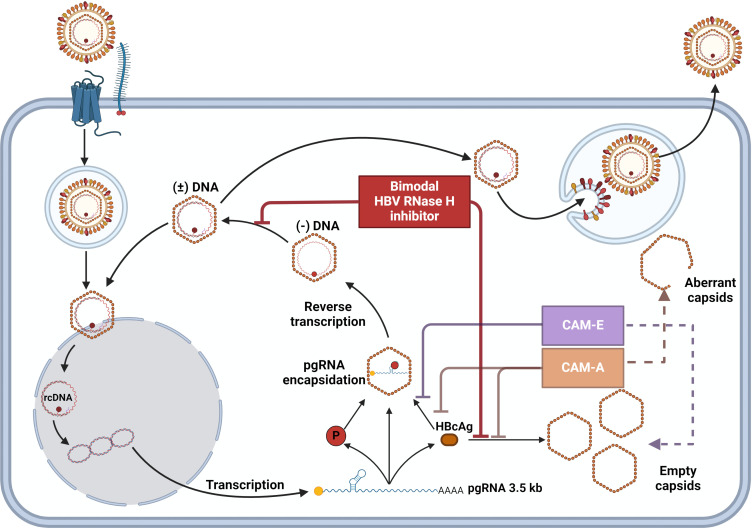
HBV replication cycle with modeled activity of 1466, a bimodal HBV RNaseH-CAM-I Inhibitor, and other small molecule inhibitors used in this study. Figure was made with Biorender.com.

HPDs, including **1466,** are potent HBV RNaseH inhibitors that preferentially inhibit synthesis of the HBV positive-polarity DNA strand (S1 Fig). As part of our preclinical development and evaluation of HBV RNaseH inhibitors, we aimed to define their effects on each step of HBV replication and found that HPD compounds suppress capsid accumulation in HepDES19 and HepG2.2.15 cells (Figs 1–2, and 5). Detailed analysis of the representative HPD compound **1466** revealed that it inhibits empty capsid accumulation in a dose-dependent manner independently of HBV DNA replication and when HBc is expressed by itself in cells (Fig 4). Finally, **1466** moderately inhibits capsid assembly in biochemical Cp149 capsid assembly reactions (Fig 7). Fig 8 displays the action of bimodal HBV RNaseH-capsid assembly inhibitors in context of the HBV replication cycle.

These data establish **1466** as the type member of a new class of bimodal HBV RNaseH-capsid assembly inhibitors. **1466** slows the rate of capsid assembly in biochemical assays (Fig 7A), whereas all other known CAMs accelerate capsid assembly [17,18]. Table 1 summarizes the key differences among the classes of CAMs. Therefore, we propose the use of the acronym CAM-I (*Inhibitor*) to represent this new class of HBc-targeting inhibitors in keeping with the current naming system [18].

**Table 1:** Differences among the classes of CAMs.

	CAM-A	CAM-E	CAM-I
Capsid Assembly	Induces aberrant capsid assembly	Induces empty capsid assembly	Inhibits capsid assembly
Mechanism of action	Stabilizes dimer:dimer interactions, accelerates capsid assembly	Stabilizes dimer:dimer interactions, accelerates capsid assembly	Unknown: Possible inhibitor of capsid nucleation
Effects on HBV replication	Blocks pgRNA encapsidation	Blocks pgRNA encapsidation	RNaseH inhibition; alters capsid levels
Effects on nucleocapsids	Decrease	Decrease	No effect
Effects on empty capsids	Decrease	Increase	Decrease

Capsid assembly progresses through a series of high energy nucleation states [42–46]. Classically, a nucleus is the first stable intermediate in an assembly reaction whose rate of formation controls the rate of product formation and after which subsequent assembly is energetically downhill [47]. In HBV, there are several metastable steps between the first stable species, a pentamer of dimers [44,46], and the ultimate nucleus, which contains approximately 30 dimers [42,45]. For **1466** to affect the kinetics of assembly without having a significant effect on the final yield of capsids suggests the hypothesis that **1466** exerts its effect on capsid nucleation. The selective effect of **1466** on empty capsids also suggests that it somehow discriminates between nucleation events triggered by pgRNA:P complexes that result in nucleocapsids and those triggered by other mechanisms that yield empty capsids. This implies a structural and/or kinetic difference in the nucleation assemblages formed with and without the pgRNA:P complex.

To our knowledge, **1466** is the first identified inhibitor of HBV capsid assembly (distinct from the assembly agonism of CAMs) with specific effects in both biochemical and cellular-based assays. Only the non-specific molecule bis(ANS), a fluorescent probe routinely used to bind hydrophobic regions of proteins, has been identified as an inhibitor of capsid assembly [48]. **1466** shares no structural similarities to bis(ANS) and does not induce the formation of large protein aggregates (Fig 7B).

Empty capsids are routinely formed during HBV replication, and about 90% of all capsids and virions are empty [49,50]. **1466** reduced capsid accumulation by >80% without affecting nucleocapsid levels (Fig 5), implying that it efficiently blocks empty capsid formation without detectably altering nucleocapsid levels. While we did not measure empty virion levels, we anticipate that **1466** would similarly decrease formation of empty virions because intracellular empty capsids are the precursors to secreted empty virions. Synthesis of DNA during reverse transcription induces a poorly understood alteration to the structure of the HBV capsid that greatly increases the efficiency of envelopment of double-stranded DNA-containing nucleocapsids [35,51], but **1466** appears to act at an earlier stage of capsid maturation. Therefore, HPD capsid assembly inhibitors could be used as tools to dissect capsid accumulation from nucleocapsid formation and to enrich nucleocapsids for further analyses.

**1466** was more effective in the cell culture systems than in the biochemical capsid assembly reaction. Empty capsid assembly occurs in an HBc concentration-dependent manner in cells [52] and biochemical reactions [19]. In HepDES19 cells this results in the accumulation of capsids over time upon induction of HBV expression. In contrast, capsid assembly is induced *en masse* in the biochemical assay due to the rapid change in ionic strength caused by NaCl addition [42,53]. Furthermore, biochemical assays may take place at unrealistically high protein concentrations. The instantaneous formation of numerous active capsid assembly intermediates is expected to largely out-compete the moderate inhibitory effect of **1466**, leading to a smaller reduction in assembly rate than would be expected from its effects on capsid accumulation in cells. The lack of dose-responsiveness in the biochemical assays suggests that the effect is saturated at the lowest dose tested. This is likely due to the noise inherent in this sensitive assay coupled with the requirement that HBc concentration exceed a pseudo-critical concentration of assembly.

There are three limitations to this work. First, in-depth biochemical analyses defining the mechanism of action and binding site of **1466** have not yet been done. Second, these data cannot fully address why there is a quantitative difference in capsid formation in the cell culture and biochemical assays employing recombinant HBc. Third, these studies were performed in transfected hepatoblastoma cell lines replicating HBV rather than in infected cells. This was done because our studies address events in the HBV replication cycle that are faithfully represented in the cell lines. Twelve HPDs, some that are very similar to **1466**, have been demonstrated to be non-cytotoxic in primary human hepatocytes [54]. Compound **1133**, which is **1466** with a Cl atom instead of an F atom at the same position on the terminal aromatic ring, very efficiently inhibits HBV replication in infected HepG2-NTCP cells when added shortly after infection [55]. Therefore, we have no reason to anticipate that either efficacy or toxicity differences between HepDES19 cells and infected or primary cells will alter the effects reported here. These limitations are anticipated to be addressed by future studies.

Bimodal HBV RNaseH-capsid assembly inhibitors may provide additional benefits to patients compared to compounds with only one mode of action, particularly if the effects on HBc and nucleocapsids can be enhanced by chemical optimization. Indeed, an interaction of **1466** with HBc may help localize the molecule for subsequent inhibition of RNaseH activity. **1466** significantly decreases capsid and HBc levels in tandem with low micromolar efficacy in cell-based assays (Figs 1,2, and 5), even from steady-state conditions in HepG2.2.15 cells (Fig 5C), implying that **1466** may increase the degradation rate of HBc and/or capsids in addition to reducing formation of empty capsids. If so, our data imply the degradation of HBc/capsids is likely to have less effect than the inhibition of capsid assembly. HPD-induced HBc degradation may act via the proteasome and could lead to an increased presentation of HBc antigen on class I human leukocyte antigen complexes (HLA-I), increasing the recognition of cccDNA-containing hepatocytes by cytotoxic CD8 T-cells. Degradation could also proceed by an apoptotic mechanism similar to what has been reported to be induced by CAM-A compounds [21,56], and this may also alter presentation of capsid-derived epitopes by HLA-I [57]. Regardless, HPD-induced reduction of empty capsid assembly would reduce the levels of empty capsid and empty virion in patients. While empty capsids and empty virions possess no known biological function during HBV replication [17], reducing their levels would minimize any biological effects they may have. Reducing HBc levels could also modulate HBV transcription from the cccDNA because HBc is found in transcriptionally active cccDNA complexes and there is some [58] but not universally accepted [59] evidence for this being modulated during CAM treatment. These secondary effects of CAM-I compounds would not necessarily be readily evident in the cell culture replication systems that led to their identification, but if these mechanisms act to a significant level in infected people, they could amplify the efficacy of the bimodal inhibitors and contribute to establishment of a functional cure.

## Materials and methods

### Compounds

Structures of all compounds are in S1 Fig. The αHT compounds **110** and **390** were synthesized as described in [28,29]. The HNO **1073** was described in [55], compound **148** and **150** were reported in [60,61]. HNO **1562** was newly synthesized as described in S1 Data. The HPD **1133** (also called **A23**) was reported in [62]. The HPD **1736** was reported in [63]. HPDs **514**, **1463**, **1466**, **1467**, and **1740** were newly synthesized as described (S1 Data).

### Tissue culture

HepG2 cells are a human hepatoblastoma cell line purchased from American Type Culture Collection (HB-8065). HepDES19 cells are a stably transfected HepG2 cell line with an integrated tetracycline-repressible HBV (genotype D) expression cassette under control of the basal CMV immediate early promoter [25]. They were maintained in 1 µg/mL tetracycline to suppress HBV replication, and tetracycline was removed to initiate HBV replication. HepG2.2.15 is a stably transfected HepG2 cell line that constitutively expresses HBV (genotype D) from endogenous HBV promoters and produces infectious virions [64]. Cells were grown on collagen-coated plates (Corning 356450) in DMEM/F12 (Cytiva SH30023) supplemented with 10% FBS and 100 IU/mL penicillin/streptomycin at 37 °C in the presence of 5% CO_2_ at saturating humidity. Cells were used between passages 2 and 12 from thaw. All cell lines were negative for *Mycoplasma* contamination using the American Type Culture Collection Universal Mycoplasma Detection Kit (30-1012K) following the manufacturer’s instructions.

### Plasmids

pCMV-HBV-LEII is an HBV pgRNA expression vector containing a 1.2-mer genotype A HBV genome cloned into pcDNA3.1(+) that carries mutations ablating HBV surface protein synthesis preventing virion formation [37]. D702A, YMHA, and Δ-Bulge mutants in pCMV-HBV-LEII were either generated previously [37] or created using the Q5 Site-Directed Mutagenesis Kit (NEB E0554S) employing the primers in S1 Table. Identity of the mutations was confirmed by sequencing. All primers were synthesized by Integrated DNA Technologies. pcDNA 3.1-HBc is a CMV-driven HBc expression plasmid commercially generated (Genscript) that contains the genotype A HBV core ORF from pCMV-HBV-LEII.

### Transfection

Transfections employed Lipofectamine 3000 (Invitrogen L3000001) following the manufacturer’s protocol. 7 x 10^6^ HepG2 cells were seeded on a collagen-coated 10 cm plate and transfected with 18 µg of plasmid DNA in a 1:1 ratio of the indicated plasmid and a secreted embryonic alkaline phosphatase (SEAP) expression plasmid (InvivoGen psetz-seap) and incubated overnight. The next day, cells were passaged and plated on a 12-well plate at 2.5 x 10^5^ cells per well in 900 µL of media. Six hours later, cells were treated with inhibitors diluted in 100 µL of media to 10 x final concentration. Treatment was maintained for 48 hours prior to cell lysis. SEAP activity was measured in the media using the Phospha-Light SEAP Reporter Gene Assay System (Invitrogen T1015) following the manufacturer’s instructions, and results were normalized to SEAP levels to limit transfection variation.

### HBV particle assay

HepDES19 or HepG2.215 cells were seeded on 12-well plates at 2 x 10^5^ cells/well two days prior to inhibitor treatment. In the N-Acetylcysteine (NAC) cotreatment experiments, HepDES19 cells were treated with 10 mM NAC one hour prior to inhibitor treatment as done previously [31]. Cells were treated with inhibitor(s) in a 1% DMSO vehicle for 4–5 days. Cells were lysed with core prep lysis buffer (10 mM Tris-HCL pH 7.5, 1 mM EDTA pH 8.0, 0.25% NP-40, 50 mM NaCl, 8% sucrose) supplemented with 0.2% protease inhibitor cocktail (P8340 Sigma). Cellular debris was removed by centrifugation, supernatants were supplemented with 10 mM CaCl_2_ and treated with 5 U/μL of micrococcal nuclease (New England BioLabs [NEB] M0247S) for one hour. Debris was removed by centrifugation, and the nuclease was inactivated with 20 mM EDTA pH 8.0. Lysate protein concentration was measured by Bradford assay (BioRad 5000006) following the manufacturer’s instructions. Protein-standardized lysates (20–30 µg per experiment) were resolved on 1.5% agarose gel overnight at 0.8 V/cm and transferred onto a nitrocellulose membrane (Cytiva 10600013) by capillary transfer using 10x SSC (1.5 M NaCl, 0.17 M sodium citrate, pH 7.0) as previously described [26]. Membranes were blocked after transfer with 1% nonfat dry milk (NFDM) in 1X Tris-buffered saline with 0.1% Tween 20 (TBST) for 1 hour, then incubated overnight at 4 °C with a mouse anti-HBV core primary antibody (Tokyo Future Style 2AHC24, clone T2221). Membranes were washed with TBST/NFDM and incubated with an HRP-linked anti-mouse IgG secondary antibody (Cell Signaling Technology 7076S) for two hours. Capsid accumulation was detected using the SuperSignal West Pico PLUS Chemiluminescent Substrate (ThermoFisher 24580) and imaged by film or Bio-RAD ChemiDoc Imaging System (Bio-Rad #12003153).

### Western blot

Cytoplasmic lysates containing 20–30 µg protein were heated at 95 °C for 10 minutes in the presence of SDS-PAGE loading buffer (0.1 M Tris-HCl pH 6.8, 10% glycerol, 1% SDS, 0.05% bromophenol blue, 5% β-mercaptoethanol). Samples were resolved on a 15% SDS-PAGE gel (10 V/cm) using a Tris-Glycine-SDS running buffer (25 mM Tris pH, 0.192 mM glycine, 0.5% SDS). Samples were blotted onto a PVDF membrane (Millipore Sigma IPVH85R) by wet tank transfer (25 mM Tris pH 8.0, 0.192 mM glycine, 15% methanol, 0.1% SDS) (30 V/cm) for 30 mins at 4 °C. Membrane development and detection followed the same protocol as the HBV capsid assay employing the same antibodies. Membranes were then stripped using a mild stripping buffer (Abcam) and re-probed with a β-actin specific antibody (Millipore Sigma MAB1501). Capsid and HBc accumulation were quantified by densitometry using FIJI’s lane select tool [65]. Technical replicates from two independent vehicle control wells in each assay were averaged and used to normalize the experimental samples.

### Encapsidated HBV pgRNA quantification

Cytoplasmic lysates were treated with micrococcal nuclease as described in the HBV particle assay to degrade non-encapsidated RNA and DNA. Lysates were diluted to 1 μg/μL protein in 50 μL. Three volumes of Trizol (Sigma-Aldrich T3935) were added and vortexed to release encapsidated RNA. Trizol extraction was performed following the manufacturer’s instructions. Nucleic acid samples were treated with DNase I (NEB M0303) and extracted once with phenol-chloroform. cDNA was synthesized using the ProtoScript First Strand cDNA Synthesis Kit (NEB E6300) using random hexamer primers. HBV cDNA was quantified by TaqMan quantitative PCR (qPCR) using an HBc ORF-specific primer-probe set (S2 Table) and a standard curve of an HBV monomer-containing plasmid. Cellular RNA carry-over detection (GAPDH) and non-reverse transcribed controls were measured for each sample.

### Cytoplasmic 3.5 kb RNA quantification

Total cellular RNA was extracted via Trizol following the manufacturer’s instructions. Samples were treated with DNase I, cDNA was synthesized, and RT-qPCR was done as described above. Relative 3.5 kb RNA levels were measured by the 2^−ΔΔ*CT*^ method using primer-probe sets specific for HBV core ORF and GAPDH (S2 Table).

### qPCR

qPCR reactions employed the Kappa Probe Force universal PCR master mix (Roche) using a QuantStudio 5 Real-Time PCR system (ThermoFisher) for 45 cycles of 95 °C for 15 s and 60 °C for 1 minute, containing 300 nM of each primer and 100 nM of the probe described in S2 Table. All primers and probes were synthesized by Integrated DNA Technologies.

### Recombinant capsid assembly reaction

Recombinant HBc Cp149 was purified, and capsid assembly was induced as previously described [19]. HBc Cp149 was mixed with a titration of **1466** prior to the addition of NaCl to 300 mM to initiate capsid assembly. Capsid assembly was measured by light scattering at 310 nm. Capsids assembled in the presence of **1466** were negatively stained and visualized by transmission electron microscopy.

### Data analysis

Data were analyzed in GraphPad Prism using the statistical methods indicated; p ≤ 0.05 indicates significance. 50% effective concentrations (EC_50_) for capsid accumulation were determined using GraphPad Prism’s 4-parameter dose-response algorithm.

## Supporting information

S1 Fig Compound structures and preferential inhibition of the HBV plus-polarity DNA strand. Structures and average EC_50_ and CC_50_ values from two to five independent assays are shown. Also shown is a representative replication inhibition experiment with three day compound exposure in HepDES19 cells. DNA levels for the plus- and minus-polarity strands were quantified by strand-preferential qPCR from the same stock of DNA and the data were normalized to vehicle-treated samples from the same 96-well plate.
(TIF)

S2 Fig HPD compounds 1736 and 1740 also suppress accumulation of HBV capsids. HepDES19 cells were treated with 1 or 10 µM of the indicated HPD compounds for five days and HBV capsids in cellular lysates were detected by HBV particle assay.
(TIF)

S3 Fig αHT compound 390 does not affect intracellular capsid accumulation or encapsidated HBV RNA levels in HepG2.2.15 cells. HBV transgenic HepG2.2.15 cells that constitutively express HBV from its native promoters were treated with a titration of **390** for four days with 20 µM **LAM** cotreatment, and intracellular capsids were detected by HBV particle assay and encapsidated HBV RNAs were detected by RTqPCR.
(TIF)

S4 Fig 1466 does not induce disassembly of pre-formed HBV capsids in cell lysates. HepDES19 cells replicating HBV were lysed, compound was added at 10 µM, samples were incubated at 37 °C, and aliquots of the lysates were removed at the indicated times. Capsids were then detected in the lysates by HBV particle assay.
(TIF)

S5 Fig 1466 does not change total yield of capsids during biochemical assembly reactions. Cp149 assembly reactions were conducted in presence of the indicated concentrations of **1466**, the reactions were allowed to reach equilibrium, and then the products were resolved by size exclusion chromatography on a Superose 6 column. Capsids eluted at ~ 9 ml and dimers eluted at ~ 18 ml. *Top panel*: Elution profiles. *Bottom panel*: The proportion of Cp149 in the dimer peak was plotted as a function of **1466** concentration. Error bars are 1 standard deviation.
(TIF)

S1 Data Chemical synthesis documentation. (DOCX)

S2 Data Original data images. (PDF)

S1 Table Primers used for mutagenesis. (DOCX)

S2 Table Primers used for RT-qPCR. (DOCX)
