# Supplementary figures and images for: Discovery of bimodal hepatitis B virus ribonuclease H and capsid assembly inhibitors
Source: PLoS Pathog. 2025 Feb 10;21(2):e1012920. doi: 10.1371/journal.ppat.1012920 (PMC11828405; doi:10.1371/journal.ppat.1012920)

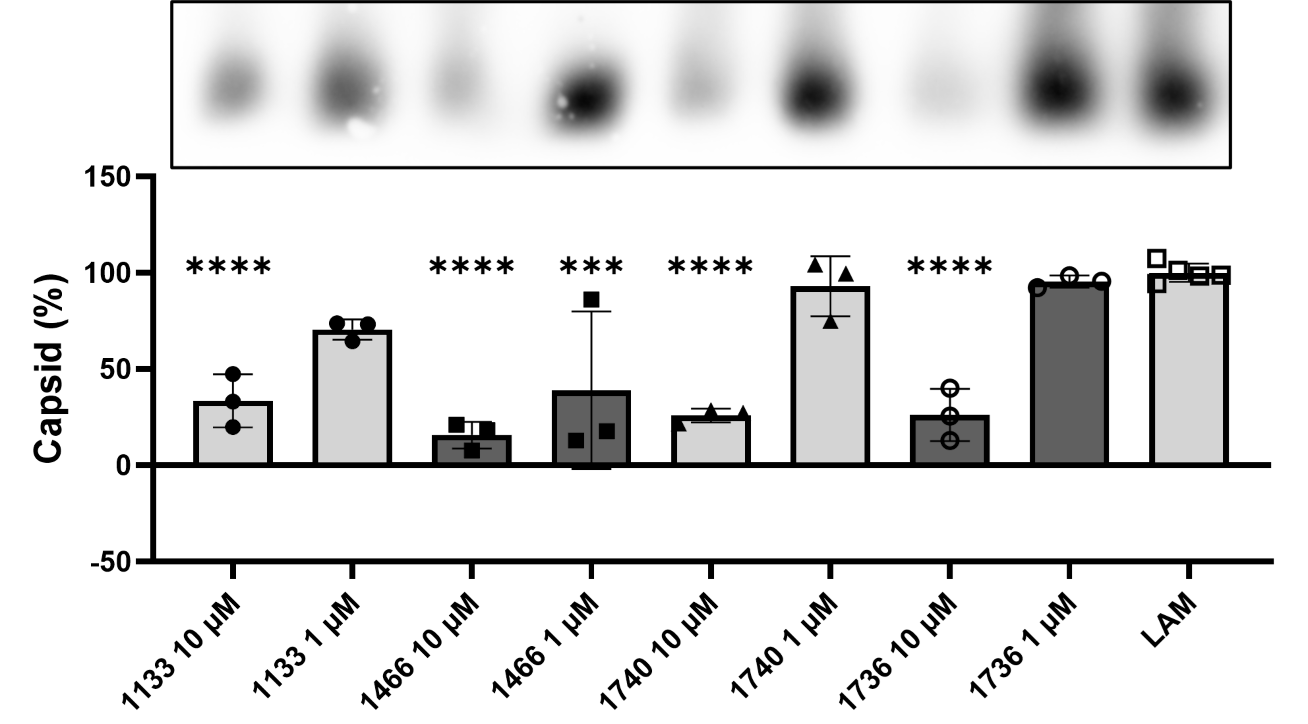

Supplement: S2 Fig — HepDES19 cells were treated with 1 or 10 µM of the indicated HPD compounds for five days and HBV capsids in cellular lysates were detected by HBV particle assay. (TIF) [file ppat.1012920.s002.tif]

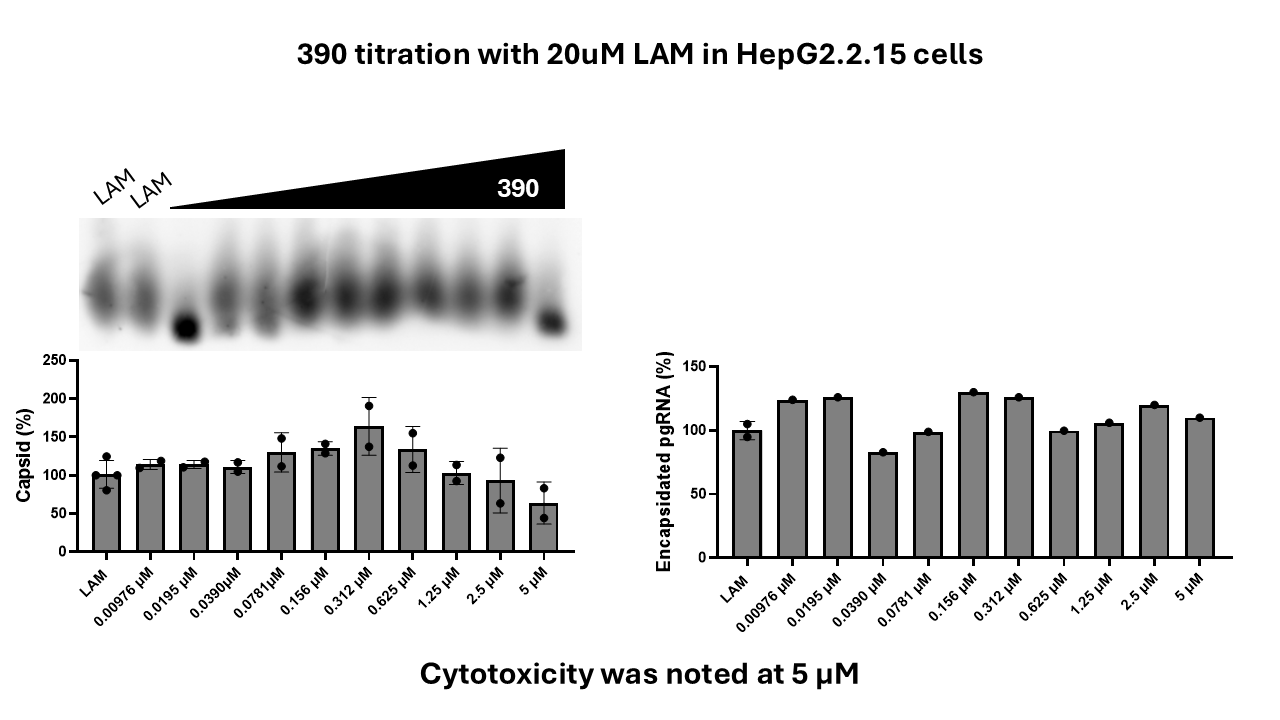

Supplement: S3 Fig — HBV transgenic HepG2.2.15 cells that constitutively express HBV from its native promoters were treated with a titration of 390 for four days with 20 µM LAM cotreatment, and intracellular capsids were detected by HBV particle assay and encapsidated HBV RNAs were detected by RTqPCR. (TIF) [file ppat.1012920.s003.TIF]

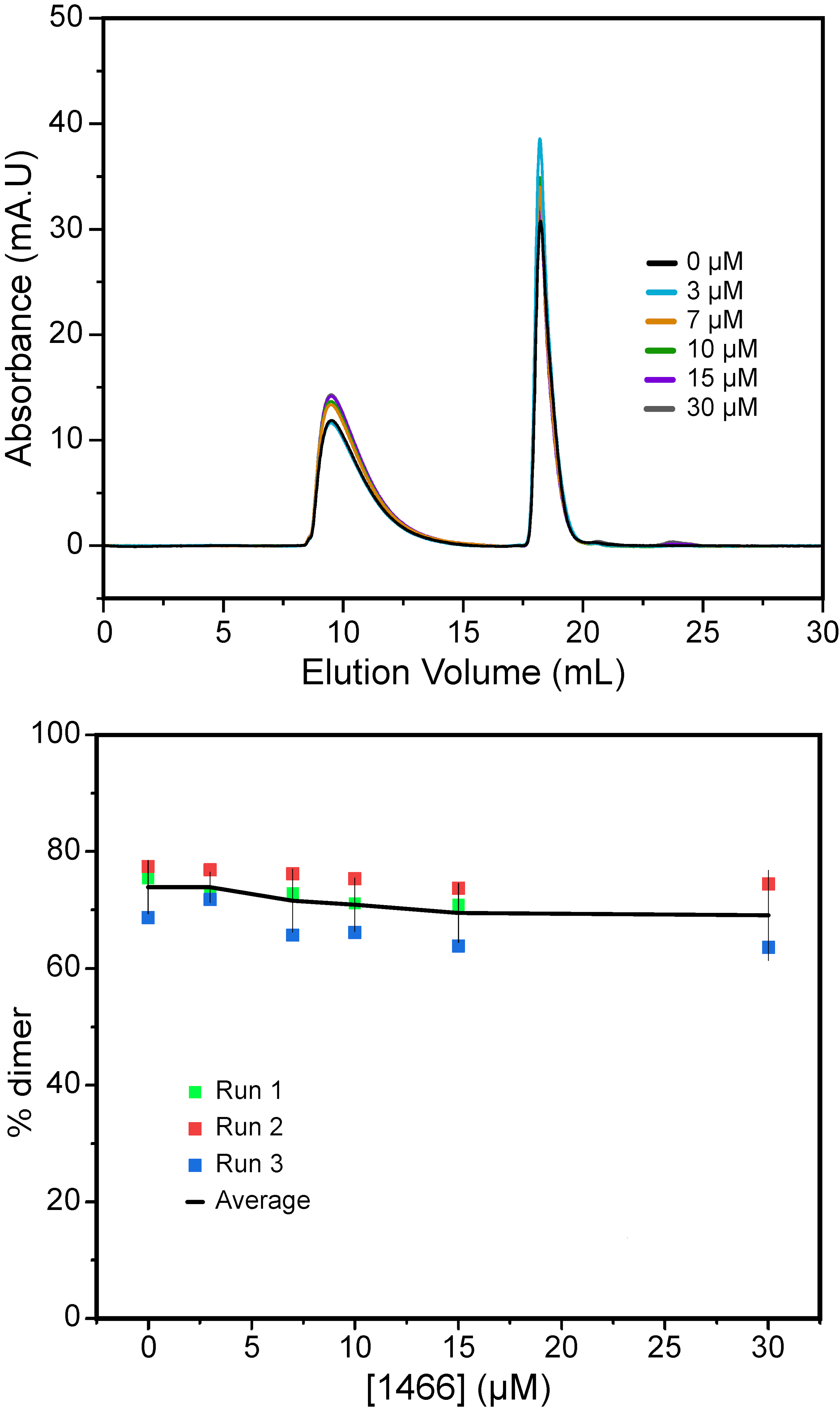

Supplement: S5 Fig — Cp149 assembly reactions were conducted in presence of the indicated concentrations of 1466, the reactions were allowed to reach equilibrium, and then the products were resolved by size exclusion chromatography on a Superose 6 column. Capsids eluted at ~ 9 ml and dimers eluted at ~ 18 ml. Top panel: Elution profiles. Bottom panel: The proportion of Cp149 in the dimer peak was plotted as a function of 1466 concentration. Error bars are 1 standard deviation. (TIF) [file ppat.1012920.s005.tif]
