# Supplementary material for: Discovery of bimodal hepatitis B virus ribonuclease H and capsid assembly inhibitors
Source: PLoS Pathog. 2025 Feb 10;21(2):e1012920. doi: 10.1371/journal.ppat.1012920 (PMC11828405; doi:10.1371/journal.ppat.1012920)
Supplement: S1 Data — (DOCX) [file ppat.1012920.s006.docx]

**HNO compounds**

**General Procedure**. Unless otherwise stated, all reagents and solvents purchased were used as received without further purification. Microwave reactions were performed on a Biotage Initiator microwave using standard Biotage microwave vessels. Normal and reverse-phase chromatography were performed using CombiFlash® Rf+ (Teledyne Isco) with SiliaFlash F_60_ 40−63 μm (230−400 mesh) silica gel (SiliCycle Inc.) eluting with hexanes/ethyl acetate gradient and RediSep Rf Gold pre-packed C18 cartridge eluting with water/acetonitrile gradient, respectively. Thin layer chromatography was carried out using TLC Silica gel 60 F_254_ Glass plates 2.5 x 7.5 cm (Merck KGaA) and visualized with a UV lamp (254/365nm UV/6-watt, BioGrow®). Liquid chromatography mass spectrometry (LCMS) was performed with an Agilent 1100 HPLC/MSD electrospray mass spectrometer in positive ion mode with a scan range of 100−1000 Da. ^1^H and ^13^C NMR spectra of intermediates and final compound were recorded and acquired in CDCl_3_ or DMSO-*d*_6_ as solvents using Bruker 400 MHz spectrometer at ambient temperature (400 MHz for ^1^H, and 100 MHz for ^13^C). Chemical shifts for ^1^H NMR (400 MHz) spectra are reported in parts per million (ppm) from either CDCl_3_ (7.26 ppm) or DMSO-*d*_6_ (2.50 ppm) with multiplicity (s = singlet, bs = broad singlet, d = doublet, t = triplet, q = quartet, and m = multiplet) and coupling constants (*J*) in Hz. Chemical shifts for ^13^C NMR (100 MHz) spectra are reported in parts per million (ppm) from either CDCl_3_ (77.2 ppm) or DMSO-*d*_6_ (39.52 ppm). High resolution mass spectrum (HRMS) was obtained with an ABSciex 5600+ instrument. The verified purity of final compound was ≥95% as determined by HPLC UV absorbance.

**Synthetic Scheme**

**Ethyl 6-(benzo[*d*][1,3]dioxol-5-yl)-1-(benzyloxy)-4-hydroxy-2-oxo-1,2-dihydro-1,8-naphthyridine-3-carboxylate (2)**. Ethyl 1-(benzyloxy)-6-bromo-4-hydroxy-2-oxo-1,2-dihydro-1,8-naphthyridine-3-carboxylate (**1**)^1^ (500 mg, 1.19 mmol), 2-(benzo[*d*][1,3]dioxol-5-yl)-4,4,5,5-tetramethyl-1,3,2-dioxaborolane (355 mg, 1.43 mmol), potassium carbonate (412 mg, 2.18 mmol), and Pd(dppf)Cl_2_ (124 mg, 0.167 mmol) were added to a microwave vial which was capped and evacuated under vacuum and backfilled with argon three times. Water (6 mL) and DMF (24 mL) were added. The mixture was heated in the microwave at 110 ^o^C for 30 min. The resultant mixture was filtered through a pad of silica gel to give the crude title compound. LC-MS: m/z 461 (M+H)^+^.

**6-(benzo[*d*][1,3]dioxol-5-yl)-1,4-dihydroxy-1,8-naphthyridin-2(1*H*)-one (Compound 1562)**. Crude intermediate **2** (504 mg, 1.10 mmol) was dissolved in 3 mL water before adding 6 mL of 33% HBr in acetic acid at room temperature. The reaction flask was then heated to 80 ^o^C with stirring for 2 h. After this time, the reaction mixture was concentrated on a rotary evaporator. The resulting solids were dissolved in 2 M sodium hydroxide and heated to 130 C for 2 h. The reaction was cooled to room temp, placed in an ice bath, and treated with conc. HCl until a pH of 2 was reached. The resulting solids were filtered and purified via reverse-phase chromatography (0→100%, CH_3_CN/H_2_O/0.1% formic acid) to afford the desired product as an off-yellow powder (21.2 mg, 6%). ^1^H NMR (400 MHz, DMSO-*d*_6_) δ ppm 10.68 (br. s., 4H), 8.91 (br. s., 1H), 8.33 (br. s., 1H), 7.39 (br. s., 1H), 7.24 (d, J = 6.36 Hz, 1H), 7.04 (d, J = 6.36 Hz, 1H), 6.08 (br. s., 2H), 5.95 (br. s., 1H). LC-MS (ESI-): HPLC >95% purity; m/z 297 (M-H). HRMS (ESI+) m/z: [M+H]^+^ Calcd for C_15_H_10_N_2_O_5_ 299.0668; found 299.0671.

**^1^H NMR Spectra of Compound 1562 in DMSO-*d*_6_:**


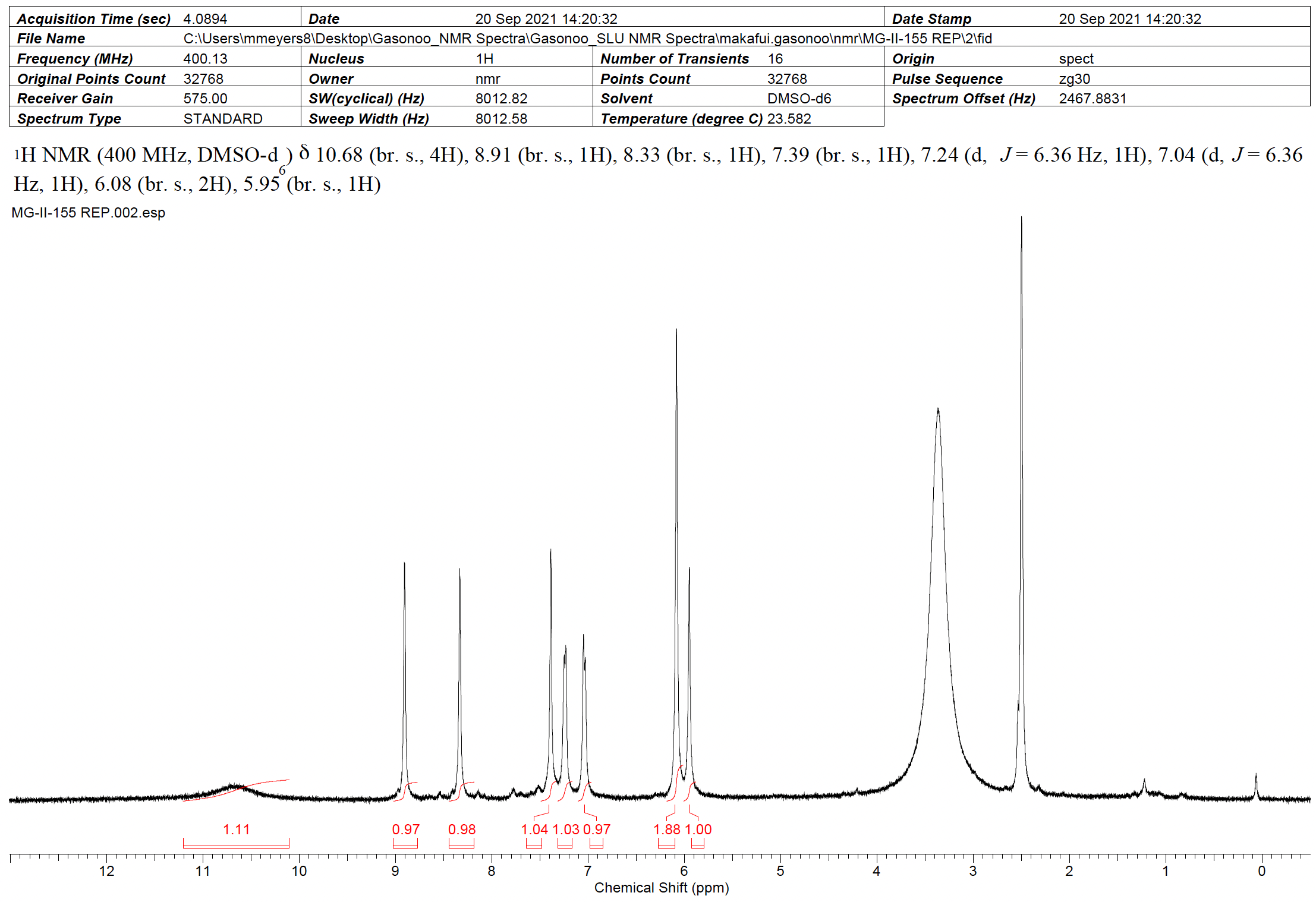


**HPLC spectra of compound 1562**


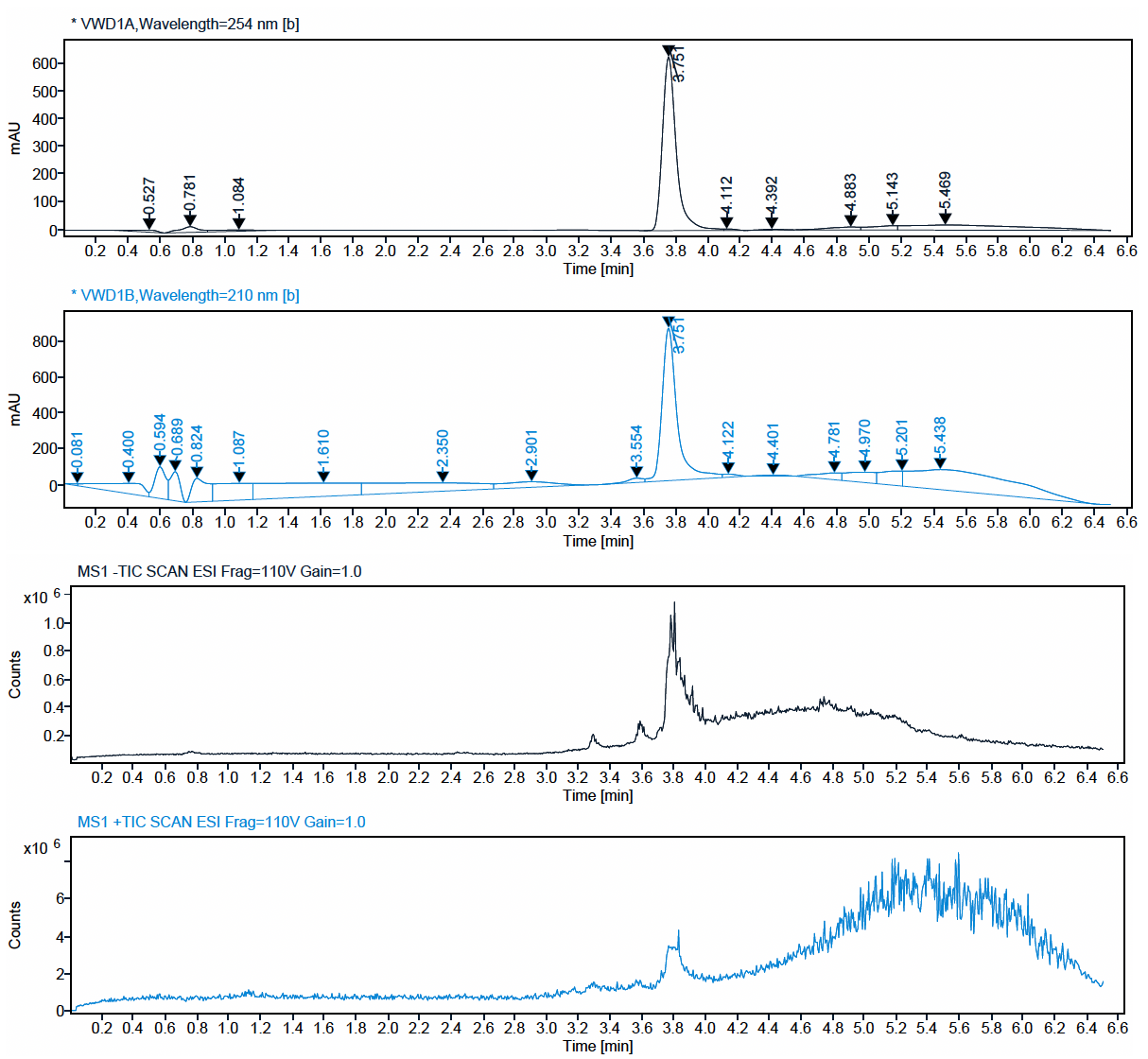


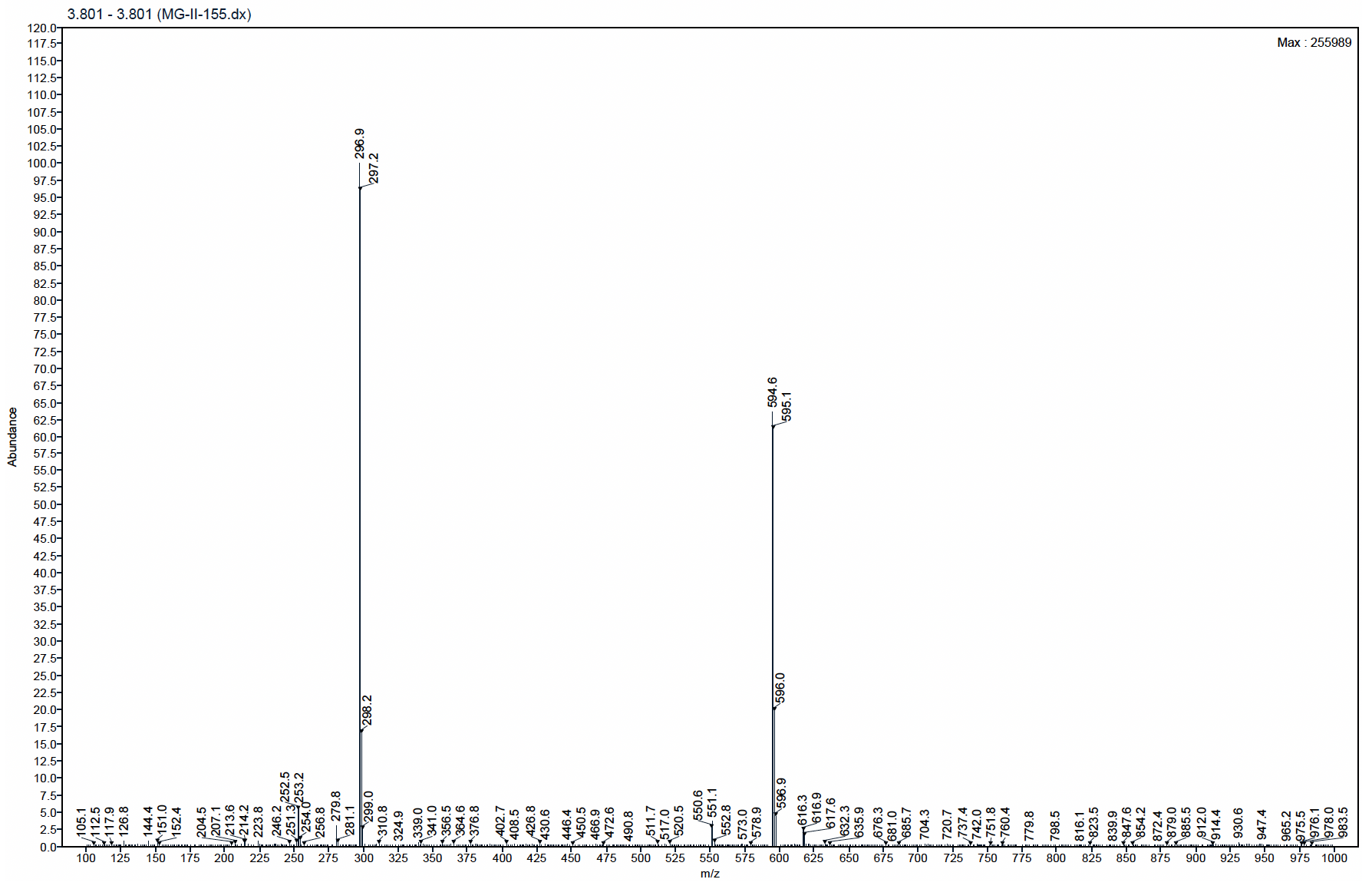


**HRMS spectra of compound 1562**

**References**

(1) Williams, P. D.; Venkatraman, S.; Langford, H. M.; Kim, B.; Booth, T. M.; Grobler, J. A.; Staas, D.; Ruzek, R. D.; Embrey, M. W.; Wiscount, C. M.; Lyle, T. A. 1-Hydroxynaphthyridine Compounds as Anti-HIV Agents. WO2008/010964 A1.

**HPDs and related compounds**

**Scheme 1.** Synthesis of compounds.

**Synthesis of 5-Acetyl-1,6-dihydroxy-4-methylpyridin-2(1*H*)-one (Β)**

To produce 5-Acetyl-1-(benzyloxy)-6-hydroxy-4-methylpyridin-2(1*H*)-one (**A**), a stirred solution of *O*-benzylhydroxylamine (2.98 g, 24.2 mmol, 1.0 equiv.) and triethylamine (2.45 g, 3.38 mL, 24.2 mmol, 1.0 equiv.) in 19 mL dry toluene was cooled in an ice-bath, and diketene (4.07 g, 3.73 mL, 48.4 mmol, 2.0 eq) was added dropwise. After 4.5 h of stirring at 65 °C under argon, the mixture was concentrated to dryness under reduced pressure and treated with 150 mL HCl 10%. The residue was partitioned between the aqueous phase and AcOEt (300 mL), the organic phase was extracted once more with HCl 10% (150 mL), and the combined aqueous phases were extracted once more with 150 mL AcOEt. The combined organic phases were washed with brine (3 × 200 mL), dried over anhydrous Na_2_SO_4_, and the solvent was removed in vacuo. The residual brownish solid was triturated with Et_2_O and AcOEt sequentially to afford the title compound A as a beige crystalline solid (4.95 g, 75 %); m.p. 144–146 °C (MeOH, AcOEt/*n*-pentane), R*_f_* _(_*_NP-TLC_*_)_ = 0.25 (AcOEt). ^1^H NMR (600.11 MHz, DMSO-*d*_6_) δ 2.34 (s, 3H, 4-C*H*_3_), 2.60 (s, 3H, 7-C*H*_3_), 5.05 (s, 2H, C*H*_2_Ph) 5.83 (s, 1H, H_3_), 7.37–7.44 (complex m, 3H, H_3′_, H_4′_, H_5′_), 7.54 (dd, 2H, *J*_1_ = 7.5 Hz, *J*_2_ = 1.7 Hz, H_2′_, H_6′_). ^13^C NMR (50.32 MHz, DMSO-*d*_6_) δ 23.8 (4-*C*H_3_), 29.0 (7-*C*H_3_), 76.9 (*C*H_2_Ph), 104.8 (C_5_), 106.7 (C_3_), 128.2 (C_3′_, C_5′_), 128.6 (C_4′_), 129.3 (C_2′_, C_6′_), 134.9 (C_1′_), 150.5 (C_4_), 159.0 (C_2_), 164.8 (C_6_), 193.4 (C_7_). Elemental analysis calcd (%) for C_15_H_15_NO_4_: C, 65.92; H, 5.53; N, 5.13; found: C, 66.00; H, 5.59; N, 5.08 (Giannakopoulou et al., 2024).

To produce 5-Acetyl-1,6-dihydroxy-4-methylpyridin-2(1*H*)-one (**514**), a solution of **A** (2.0 g, 7.32 mmol) in 120 mL MeOH was hydrogenated for 20 min at rt and 40 psi pressure, in the presence of 200 mg Pd/C (10 wt.%) as a catalyst. The catalyst was filtered off, washed with portions of hot MeOH (3 × 20 mL) and the combined filtrates were evaporated under reduced pressure. The beige crystalline product was treated with AcOEt to yield the *N*-hydroxypyridinedione **514** almost quantitatively (1.32 g, 98 %); m.p. 178–179 °C (MeOH/*n*-pentane, dry Et_2_O), R*_f_* _(_*_NP-TLC_*_)_ = 0.06 (AcOEt), R*_f_* _(_*_RP-TLC_*_)_ = 0.85 (H_2_O/ACN 7:3). ^1^H NMR (600.11 MHz, DMSO-*d*_6_) δ 2.32 (s, 3H, 4-C*H*_3_), 2.56 (s, 3H, 7-C*H*_3_), 5.80 (s, 1H, H_3_). ^13^C NMR (100.61 MHz, DMSO-*d*_6_) δ 23.3 (4-*C*H_3_), 28.0 (7-*C*H_3_), 104.7 (C_5_), 107.6 (C_3_), 149.1 (C_4_), 157.9 (C_2_), 165.1 (C_6_), 194.3 (C_7_). Elemental analysis calcd (%) for C_8_H_9_NO_4_: C, 52.46; H, 4.95; N, 7.65; found: C, 52.51; H, 5.00; N, 7.58 (Giannakopoulou et al., 2024).

**Synthesis of *O*-Substituted *N*-Hydroxyphthalimides**

General procedure:

To a solution of *N*-hydroxyphthalimide (500.0 mg, 3.07 mmol, 1 equiv.) in anhydrous DMF (3 mL), NaH 60% w/w (1.25 equiv.) is added at 0 °C. The mixture is stirred at rt for 30 min. Thereafter, the appropriate halogenide (1.5 equiv.) is added and the reaction is stirred at rt overnight. Then, water is added, and a solid precipitate is formed. The precipitate is filtered under vacuum and washed with water and a solution of n-pentane/Et_2_O 7:3. The solid is dried over P_2_O_5_, to afford the desired product.

The compound 2-((2-chlorobenzyl)oxy)isoindoline-1,3-dione (**1**) was synthesized from 1-chloro-2-(chloromethyl)benzene according to the general procedure. Pink solid (652.6 mg, 93 %). ^1^H NMR (600 MHz, CDCl_3_) δ 7.81 (dd, *J* = 5.4, 3.1 Hz, 2H), 7.74 (dd, *J* = 5.5, 3.1 Hz, 2H), 7.65 – 7.62 (m, 1H), 7.41 – 7.38 (m, 1H), 7.33 – 7.28 (m, 2H), 5.37 (s, 2H) (Wang et al., 2011).

The compound 2-((3,5-dimethylbenzyl)oxy)isoindoline-1,3-dione (**2**) was synthesized from 1-(bromomethyl)-3,5-dimethylbenzene according to the general procedure. White solid (700.0 mg, 81 %). ^1^H NMR (400 MHz, CDCl_3_) δ 7.86 – 7.80 (m, 2H), 7.77 – 7.71 (m, 2H), 7.16 (s, 2H), 7.01 (s, 1H), 5.13 (s, 2H), 2.32 (s, 6H) (Jiang et al., 2019).

The compound 2-((4'-(Chlorobenzyl)oxy)isoindolin-1,3-dione (**3**) was synthesized from 1-(bromomethyl)-4-chlorobenzene according to the general procedure. White solid (561.2 mg, 80 %). ^1^H NMR (600 MHz, CDCl_3_) δ 7.82 (dd, J = 5.4, 3.1 Hz, 2H, Ar- Pthal), 7.74 (dd, J = 5.4, 3.1 Hz, 2H, Ar- Pthal), 7.48 (d, J = 8.4 Hz, 2H, Ar), 7.35 (d, J = 8.4 Hz, 2H, Ar), 5.18 (s, 2H, OCH_2_) (Bhargavi et al., 2017).

The compound 4-(((1,3-dioxoisoindolin-2-yl)oxy)methyl)benzonitrile (**4**) was synthesized from 4-(bromomethyl)benzonitrile according to the general procedure. Pink solid (695.3 mg, 99 %). ^1^H NMR ((400 MHz, CDCl_3_) δ 7.85 – 7.80 (m, 2H, Αr), 7.80 – 7.74 (m, 2H, Ar), 7.72 – 7.64 (m, 4H, Ar- Pthal), 5.26 (s, 2H, OCH_2_) (Wang et al., 2011).

**Synthesis of *O*-Substituted Hydroxylamines**

General procedure:

To a solution of the appropriate N-hydroxyphthalimide (250.0 mg, 1 equiv.) in CH_2_Cl_2_ (3 mL), hydrazine monohydrate 64% w/w (2 equiv.) is added, and the reaction is stirred at rt for 1–24 h. The white precipitate formed is filtered, washed with CH_2_Cl_2_, and the filtrate is concentrated to afford the corresponding hydroxylamine.

The compound O-(2-chlorobenzyl)hydroxylamine (**5**) was synthesized from the compound **1** (250.0 mg, 0.87 mmol) according to the general procedure (3 h), to afford a green oil (87.9 mg, 64 %). ^1^H NMR (400 MHz, CDCl_3_) δ 7.38-7.40 (m, 1H), 7.31-7.33 (m, 1H), 7.17-7.21 (m, 2H), 5.26 (brs, 2H), 4.76 (s, 2H) (Wang et al., 2011).

The compound O-(3,5-dimethylbenzyl)hydroxylamine (**6**) was synthesized from the compound **2** (500.0 mg, 1,78 mmol) according to the general procedure (3 h), to afford an off-yellow oil (209.7 mg, 78 %). ^1^H NMR (400 MHz, CDCl_3_) δ 6.97 (dd, *J* = 2.1, 1.1 Hz, 2H), 6.81 (t, *J* = 1.9 Hz, 1H), 5.07 (s, 2H), 4.64 (s, 2H), 2.24 (s, 6H). ^13^C NMR (125 MHz, CDCl_3_) δ 138.19, 133.91, 129.26, 126.23, 77.52, 21.07.

The compound O-(4-chlorobenzyl)hydroxylamine (**7**) was synthesized from the compound **3** (283.6 mg, 0.99 mmol) according to the general procedure (3 h), to afford an off-yellow oil (154 mg, 99 %). ^1^H NMR (400 MHz, CDCl_3_) δ 7.55 – 7.49 (m, 2H, Ar), 7.31 – 7.27 (m, 2H, Ar), 5.52 – 5.38 (m, 2H, NH_2_), 4.67 (s, 2H, OCH_2_) (Wei et al., 2013).

The compound 4-((aminooxy)methyl)benzonitrile (**8**) was synthesized from the compound **4** (263.7 mg, 0.95 mmol) according to the general procedure (3 h), to afford an off-yellow oil (139 mg, 99 %). ^1^H NMR (400 MHz, CDCl_3_) δ 7.55 – 7.49 (m, 2H, Ar), 7.31 – 7.27 (m, 2H, Ar), 5.52 – 5.38 (m, 2H, NH_2_), 4.67 (s, 2H, OCH_2_) (Bhargavi et al., 2017).

**Synthesis of *N*-Hydroxypyridinediones**

General procedure:

To a solution of the appropriate hydroxylamine (0.57 mmol, 1.05 equiv.) in abs. EtOH (2 mL), 5-acetyl-1,6-dihydroxy-4-methylpyridin-2(1*H*)-one (B) (0.55 mmol, 1 equiv.) is added and the reaction mixture is stirred at RT, under argon, overnight. Thereafter, the solvent is evaporated under vacuum. The solid residue is triturated with Et_2_O under ice to afford the desired compound as a solid.

The compound 5-(1-(((2-chlorobenzyl)oxy)imino)ethyl)-1,6-dihydroxy-4-methylpyridin-2(1*H*)-one (**1463**) was synthesized from the compound **5** (87.9 mg, 0.56 mmol) according to the general procedure. Green solid (45.0 mg, 28 %). R*_f_* = 0.06 (EtOAc/MeOH 3:1), m.p. 109-111 °C. ^1^H NMR (600 MHz, DMSO) δ 7.50 – 7.38 (m, 2H, Ar), 7.38 – 7.31 (m, 2H, Ar), 5.46 (s, 1H, H_3_), 5.18 (s, 2H, -CH_2_-), 2.04 (s, 2H, 7-CH_3_), 1.98 (s, 1H, 7-CH_3_), 1.89 (d, *J* = 8.2 Hz, 3H, 4-CH_3_). ^13^C NMR (151 MHz, DMSO) δ 156.49 (C_2_), 155.02 (C_6_), 153.91 (C_7_), 146.83 (C_2‘_), 135.92 (C_3‘_), 135.54 (C_5‘_), 132.18 (C_4‘_), 130.02 (C_1‘_), 129.93 (C_6‘_), 128.24 (C_3_), 128.06 (C_5_), 91.19 (C_4_), 60.21 (-CH_2_-), 19.51 (4-CH_3_), 15.90 (7-CH_3_). Elemental analysis calcd (%) for C_15_H_15_ClN_2_O_4_: C, 55.82; H, 4.68; N, 8.68. Found: C, 55.84; H, 4.64; N, 8.69.

The compound 5-(1-(((3,5-dimethylbenzyl)oxy)imino)ethyl)-1,6-dihydroxy-4-methylpyridin-2(1*H*)-one (**1740**) was synthesized from the compound **6** (209.7 mg, 1.39 mmol) according to the general procedure. Blue solid (177.9 mg, 45 %). R*_f_* = 0.10 (EtOAc/MeOH 3:1), mp 130-132 °C (dec.). ^1^H NMR (400 MHz, DMSO) δ 7.08 – 6.74 (m, 3H, Ar), 4.98 (dt, J = 28.2, 15.2 Hz, 2H, -CH2-), 2.39 – 2.15 (m, 6H, 3’,6’-CH3), 2.06 – 1.94 (m, 3H, 4-CH3), 1.87 (s, 3H, 7-CH3). ^13^C NMR (151 MHz, DMSO) δ 154.38 (C_6_), 152.74 (C_2_) 150.95 (C_7_), 147.33 (C_2‘, 6’_), 132.86 (C_1‘_), 131.88 (C_3‘, 5’_), 130.03 (C_4‘_), 126.76 (C_5_), 124.07 (C_3_), 78.65 (C_4_), 77.07 (-CH_2_-), 29.67 (3’,5’-CH_3_), 23.57 (7-CH_3_), 17.97 (4-CH_3_). Elemental analysis calcd (%) for C_17_H_20_N_2_O_4_: C, 64.54; H, 6.37; N, 8.86. Found: C, 64.52; H, 6.36; N, 8.88.

The compound 1,6-hydroxy-4-methyl-5-(1-(((4'-chlorobenzyl)oxy)imino)ethyl)pyridin-2(1H)-one (**1466**) was synthesized from the compound **7** (94.60 mg, 0.60 mmol) according to the general procedure. Yellow solid (122.4 mg, 69.5 %). R*_f_* = 0.10 (EtOAc/MeOH 3:1), mp 110 °C. ^1^H NMR (600 MHz, DMSO) δ 7.43 – 7.29 (m, 4H, Ar), 5.50 (s, J = 16.7 Hz, 1H, H_3_), 5.02 (s, J = 76.7 Hz, 2H, OCH_2_), 2.04 – 1.92 (s, 3H, CH_3_), 1.87 (s, J = 9.5 Hz, CH_3_). ^13^C NMR (151 MHz, DMSO) δ 156.53 (C_2_), 154.6, 154.57 (C_6_), 153.79 (C_7_), 146.8 (C_4_), 138.6 (C_1’_), 132.18 (C_4’_), 129.63 (C_2’_, C_6’_), 128.27 (C_3’_, C_5’_), 112.23 (C_5_), 91.13 (C_3_), 90.70, 73.76 (OCH_2_Ph), 23.43, 19.95 (7- CH_3_), 19.60 (4- CH_3_), 15.99 (7- CH_3_). Anal. calcd (%) for C_15_H_15_ClN_2_O_4_: C, 55.82; H, 4.68; N, 8.68. Found: C, 55.83; H, 4.70; N, 8.69.

The compound 1,6-dihydroxy-4-methyl-5-(1-(((4'-cyanobenzyl)oxy)imino)ethyl)pyridin-2(1H)-one (**1467**) was synthesized from the compound **8** (89.2 mg, 0.60 mmol) according to the general procedure. Yellow solid (135.5 mg, 79 %). R*_f_* = 0.10 (EtOAc/MeOH 3:1), mp 120 °C dec. ^1^H NMR (600 MHz, DMSO) δ 7.88 – 7.80 (m, 2H, Ar), 7.59 – 7.48 (m, 2H, Ar), 5.53 (s, 1H, H_3_), 4.78 (s, 2H, OCH_2_), 2.27 (s, J = 1.0 Hz, 3H, CH_3_), 2.48 (s, CH_3_). ^13^C NMR (151 MHz, DMSO) δ 156.53 (C_2_), 154.6, 154.57 (C_6_), 153.79 (C_7_), 146.8 (C_4_), 138.6 (C_1’_), 129.63 (C_2’_, C_6’_), 128.27 (C_3’_, C_5’_), 115.6 (C-N), 112.23 (C_5_), 108.6 (C_4’_), 91.13 (C_3_), 90.70, 73.76 (OCH_2_Ph), 23.43, 19.95 (7-CH_3_), 19.60 (4-CH_3_), 15.99 (7-CH_3_). Anal. Calcd (%) for C_16_H_15_N_3_O_4_: C, 61.34; H, 4.83; N, 13.41. Found: C, 61.36; H, 4.84; N, 13.44.

**References**

Bhargavi, M.V., Shashikala, P., Sumakanth, M., Gunda, S.K., 2017. Design, Synthesis, Molecular Docking and Biological Evaluation of Novel Coumarin-Oxime Ether Derivatives as COX-2 Inhibitors. Asian J. Chem. 29, 2559–2564. https://doi.org/10.14233/ajchem.2017.20865

Giannakopoulou, E., Pardali, V., Edwards, T.C., Woodson, M., Tajwar, R., Tavis, J.E., Zoidis, G., 2024. Identification and assessment of the 1,6-dihydroxy-pyridin-2-one moiety as privileged scaffold for HBV ribonuclease H inhibition. Antiviral Res. 223, 105833. https://doi.org/10.1016/j.antiviral.2024.105833

Jiang, H., Tang, X., Liu, S., Wang, L., Shen, H., Yang, J., Wang, H., Gui, Q.-W., 2019. Ultrasound accelerated synthesis of O-alkylated hydroximides under solvent- and metal-free conditions. Org. Biomol. Chem. 17, 10223–10227. https://doi.org/10.1039/C9OB02245G

Wang, M.-Z., Xu, H., Liu, T.-W., Feng, Q., Yu, S.-J., Wang, S.-H., Li, Z.-M., 2011. Design, synthesis and antifungal activities of novel pyrrole alkaloid analogs. Eur. J. Med. Chem. 46, 1463–1472. https://doi.org/10.1016/j.ejmech.2011.01.031

Wei, Z., Wang, J., Liu, M., Li, S., Sun, L., Guo, H., Wang, B., Lu, Y., 2013. Synthesis, in Vitro Antimycobacterial and Antibacterial Evaluation of IMB-070593 Derivatives Containing a Substituted Benzyloxime Moiety. Molecules 18, 3872–3893. https://doi.org/10.3390/molecules18043872
