# Supplementary material for: Discovery of bimodal hepatitis B virus ribonuclease H and capsid assembly inhibitors
Source: PLoS Pathog. 2025 Feb 10;21(2):e1012920. doi: 10.1371/journal.ppat.1012920 (PMC11828405; doi:10.1371/journal.ppat.1012920)
Supplement: S2 Data — (PDF) [file ppat.1012920.s007.pdf]

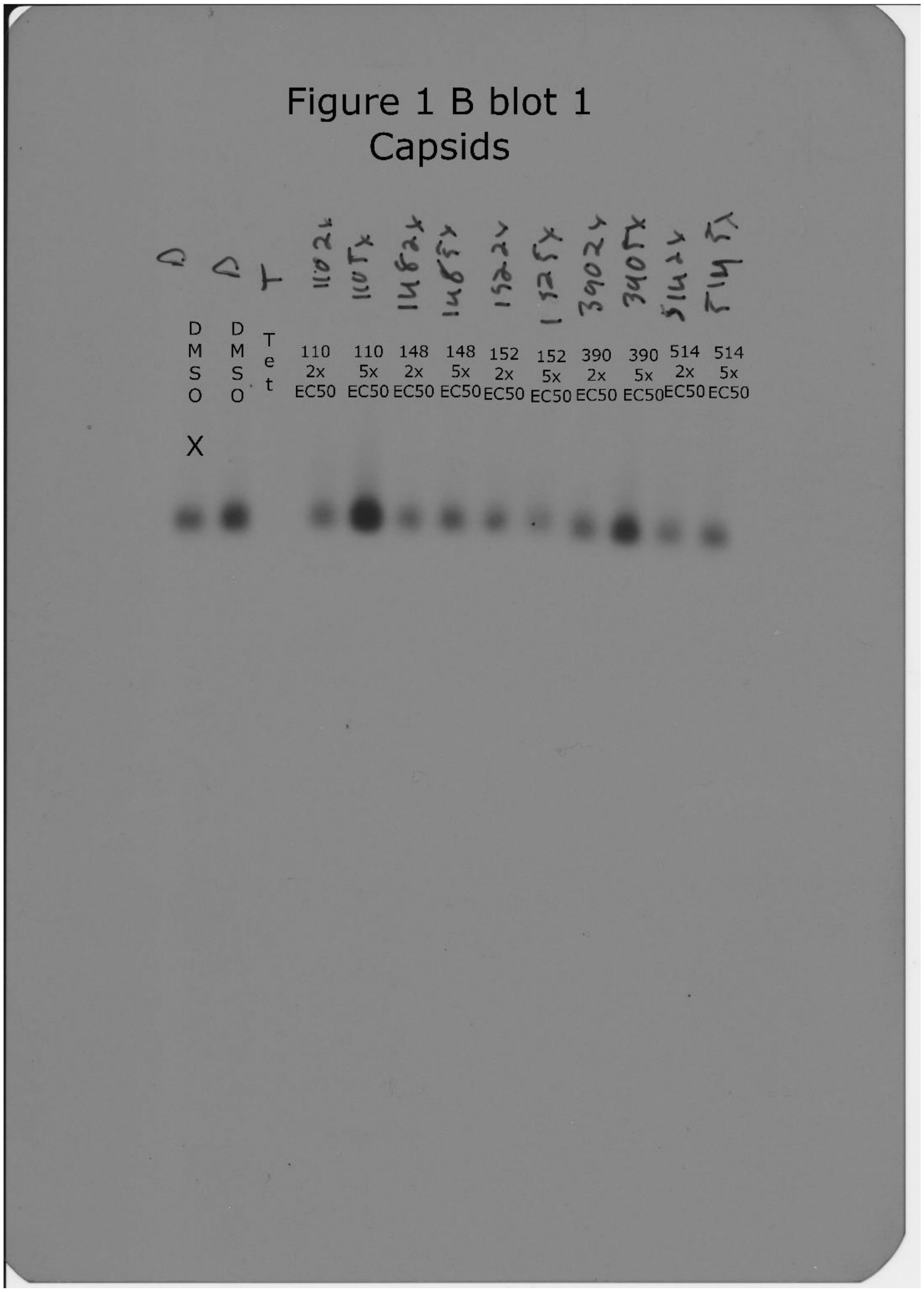
Figure 1 B blot 1
Capsids
DMSO
DMSO
Tet
110 2x EC50
110 5x EC50
148 2x EC50
148 5x EC50
152 2x EC50
152 5x EC50
390 2x EC50
390 5x EC50
514 2x EC50
514 5x EC50
X

Figure 1B Blot 2 Capsid

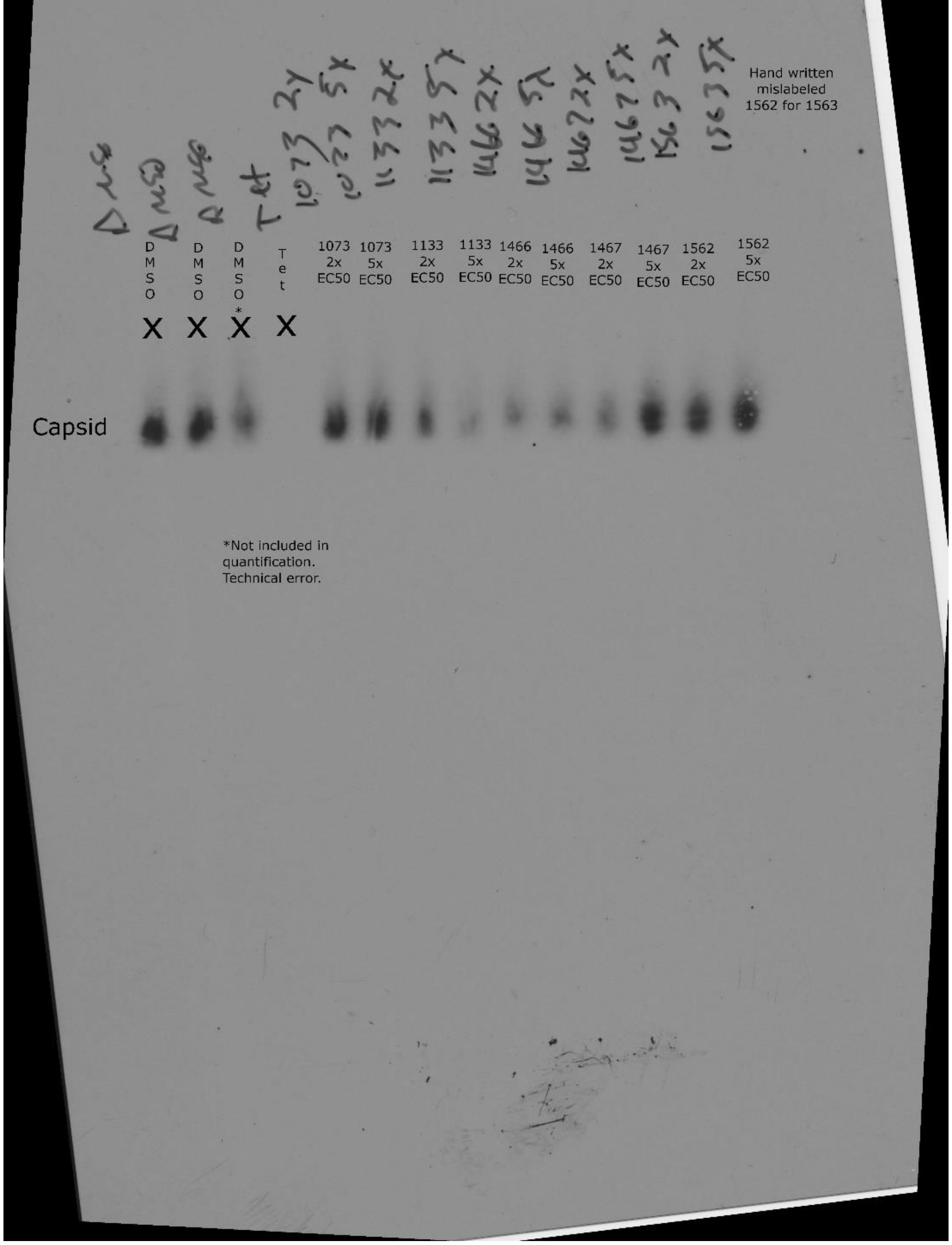

Figure 1 C Blot 1 HBc

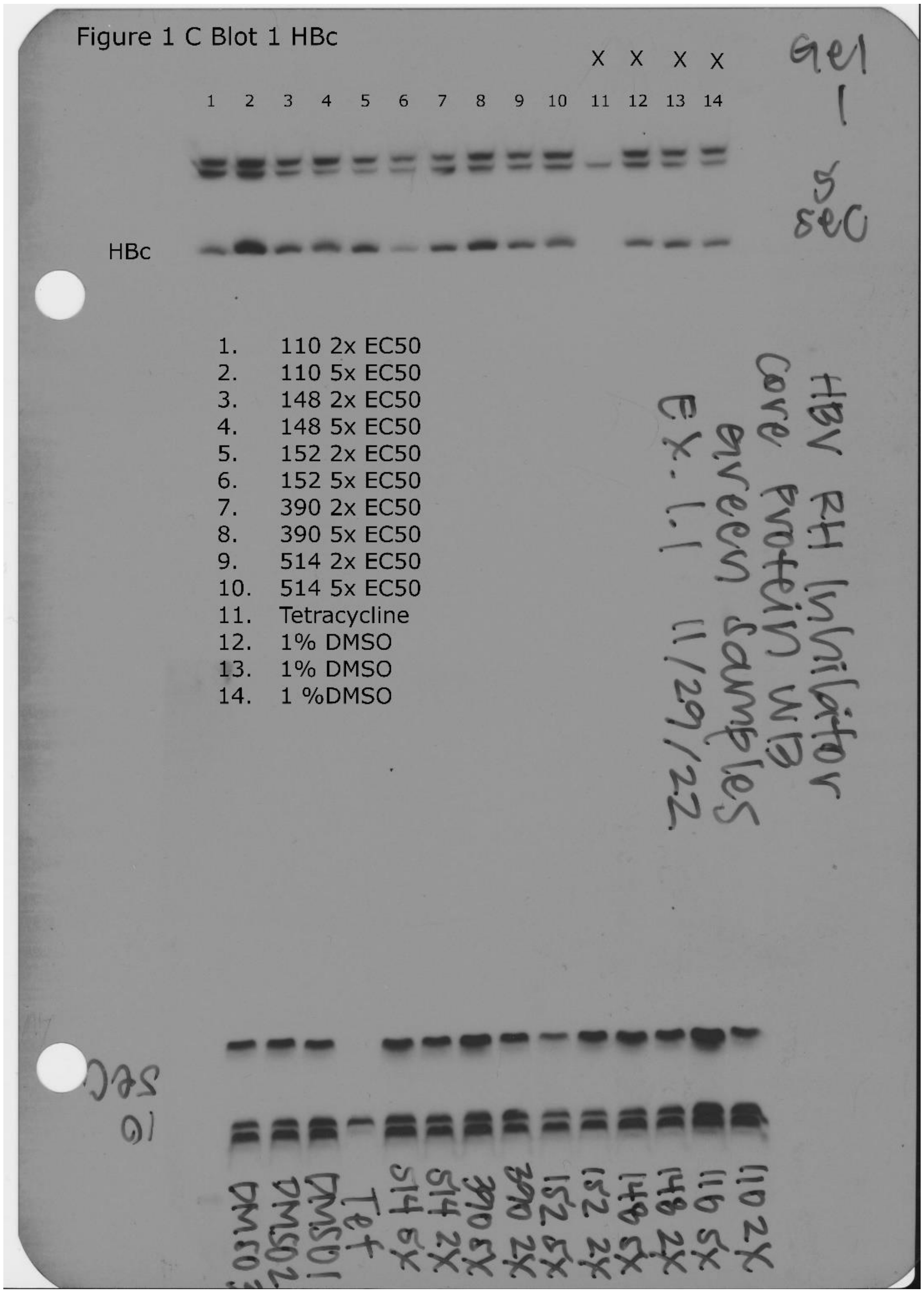

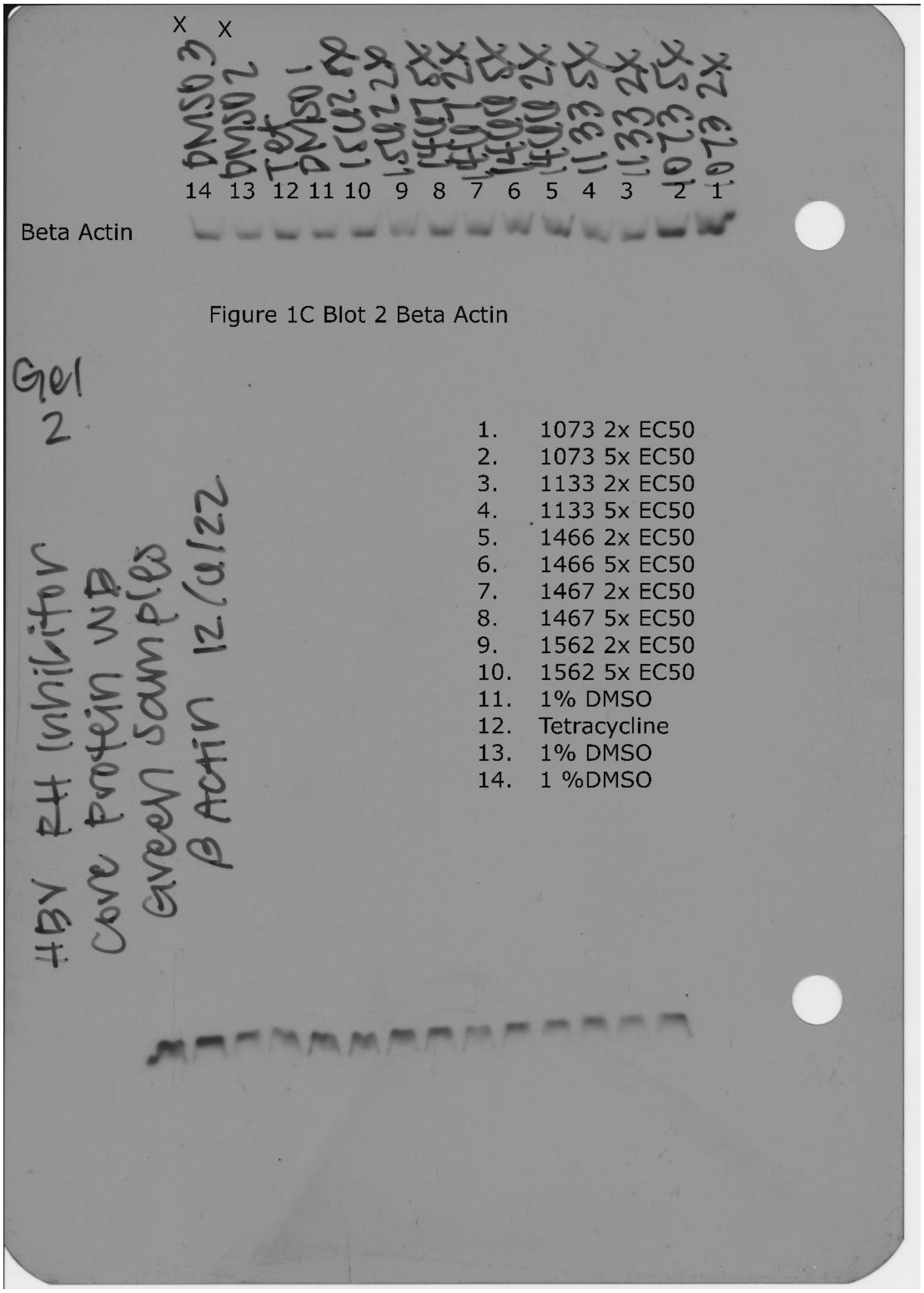
X X
14 13 12 11 10 9 8 7 6 5 4 3 2 1
Beta Actin
Figure 1C Blot 2 Beta Actin
Gel 2
1. 1073 2x EC50
2. 1073 5x EC50
3. 1133 2x EC50
4. 1133 5x EC50
5. 1466 2x EC50
6. 1466 5x EC50
7. 1467 2x EC50
8. 1467 5x EC50
9. 1562 2x EC50
10. 1562 5x EC50
11. 1% DMSO
12. Tetracycline
13. 1% DMSO
14. 1 %DMSO

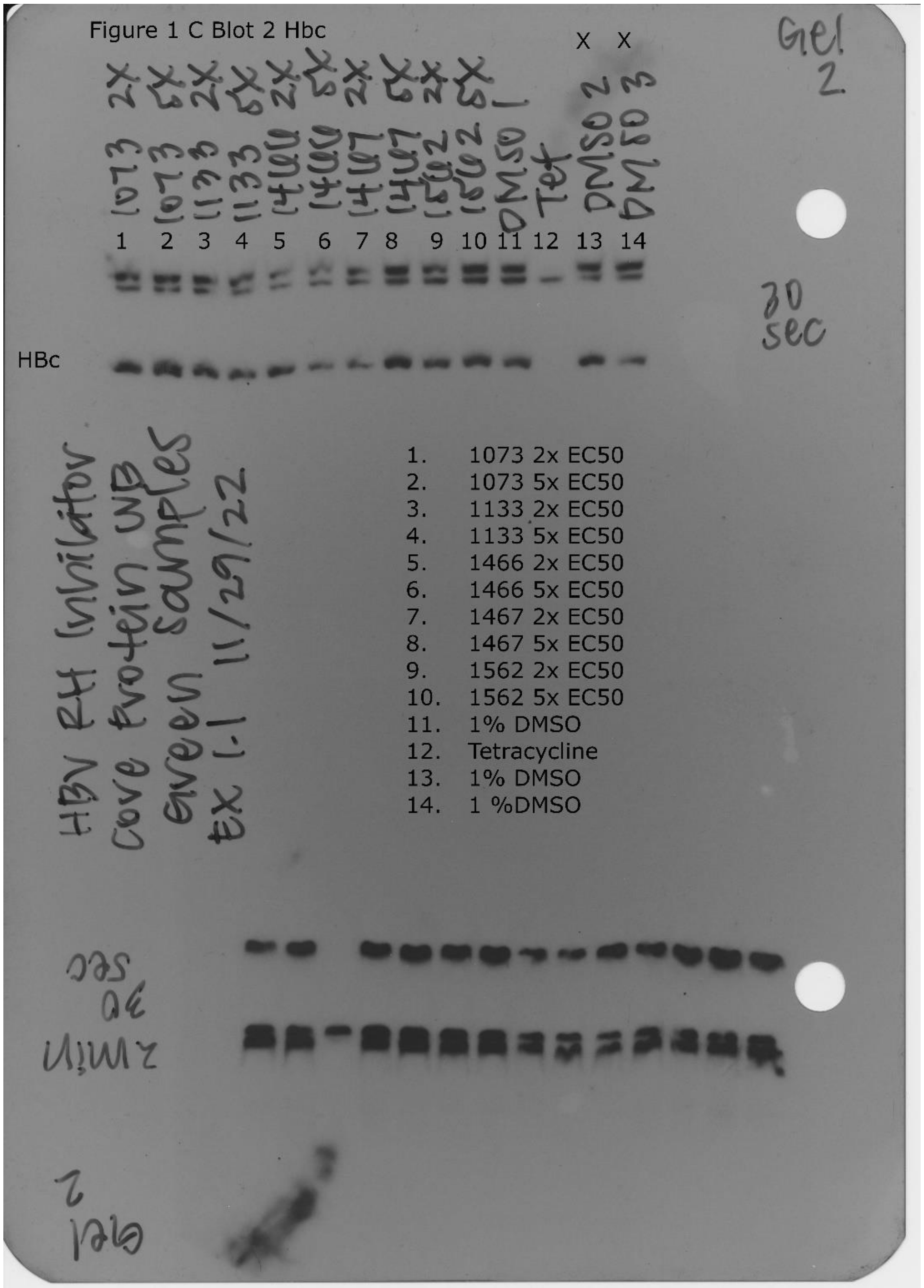

Figure 1 C Blot 2 Hbc
Gel 2
1073 2X
1073 5X
1133 2X
1133 5X
1466 2X
1466 5X
1467 2X
1467 5X
1562 2X
1562 5X
DMSO 1
Tet
DMSO 2
DMSO 3
X X
1 2 3 4 5 6 7 8 9 10 11 12 13 14
30 sec
HBc
HBV RH Inhibitor Core Protein WB Green samples EX 1.1 11/29/22
1. 1073 2x EC50
2. 1073 5x EC50
3. 1133 2x EC50
4. 1133 5x EC50
5. 1466 2x EC50
6. 1466 5x EC50
7. 1467 2x EC50
8. 1467 5x EC50
9. 1562 2x EC50
10. 1562 5x EC50
11. 1% DMSO
12. Tetracycline
13. 1% DMSO
14. 1 %DMSO

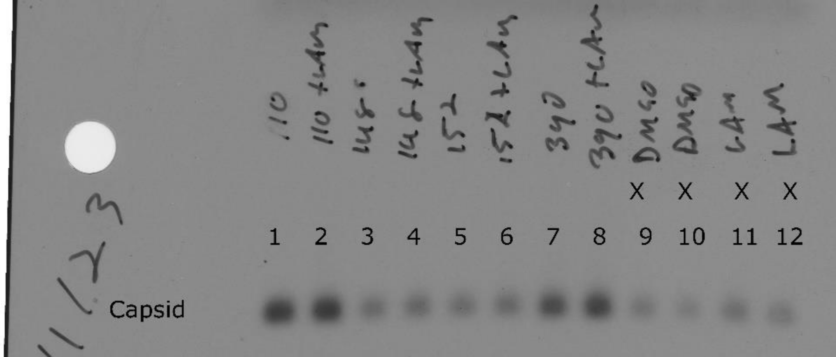

Figure 2C Blot 1 Capsid

1. 110 5x EC50
2. 110 5x EC50 + LAM
3. 148 5x EC50
4. 148 5x EC50 + LAM
5. 152 5x EC50
6. 152 5x EC50 + LAM
7. 390 5x EC50
8. 390 5x EC50 + LAM
9. 1% DMSO
10. 1% DMSO
11. 20 uM LAM
12. 20 uM LAM

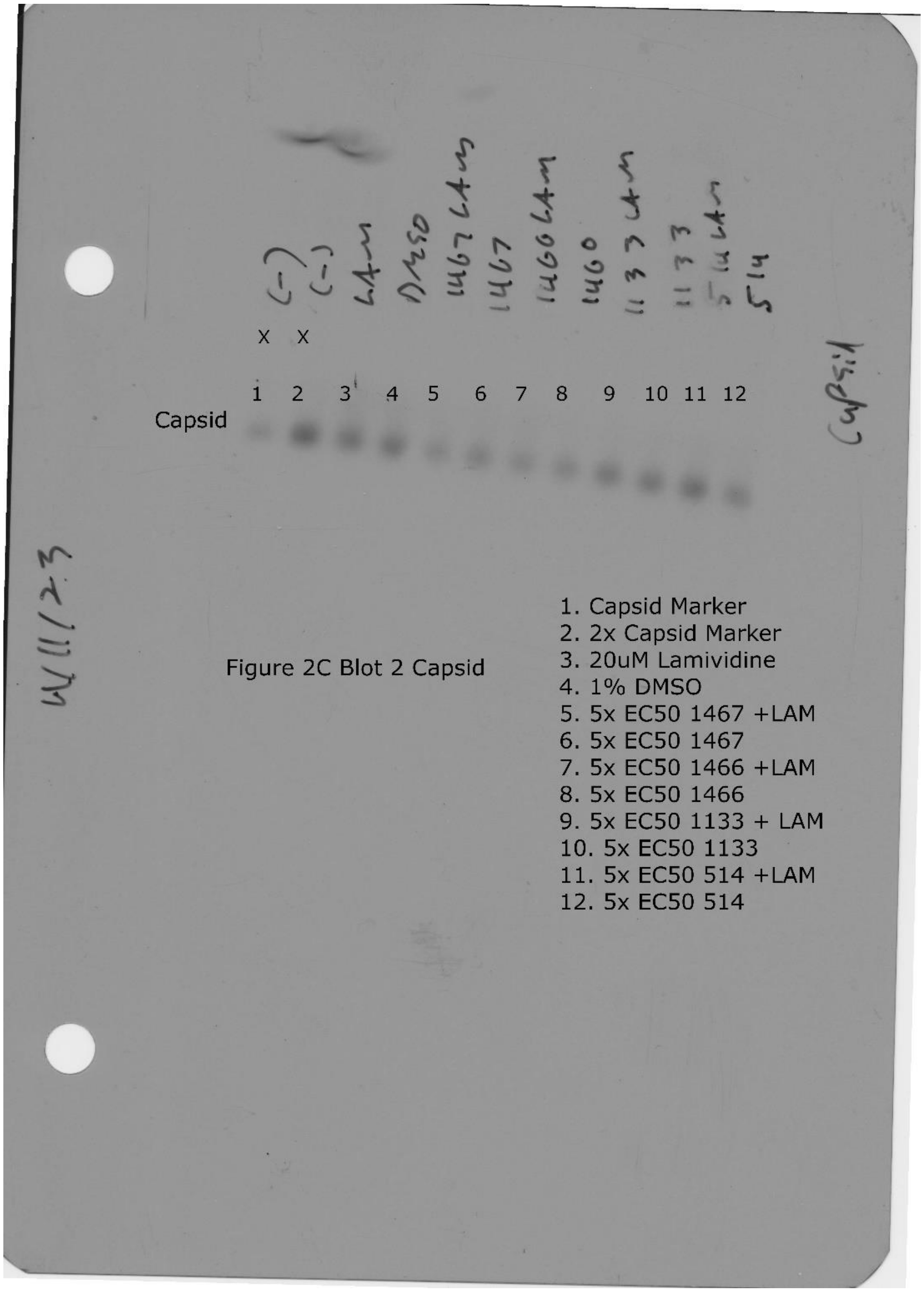
X X
1 2 3 4 5 6 7 8 9 10 11 12
Capsid
Figure 2C Blot 2 Capsid
1. Capsid Marker
2. 2x Capsid Marker
3. 20uM Lamividine
4. 1% DMSO
5. 5x EC50 1467 +LAM
6. 5x EC50 1467
7. 5x EC50 1466 +LAM
8. 5x EC50 1466
9. 5x EC50 1133 + LAM
10. 5x EC50 1133
11. 5x EC50 514 +LAM
12. 5x EC50 514

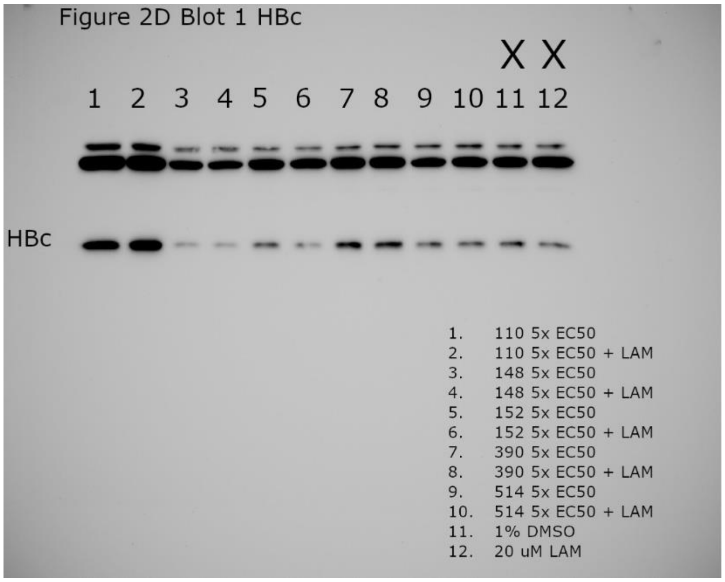
Figure 2D Blot 1 HBc
X X
1 2 3 4 5 6 7 8 9 10 11 12
HBc
1. 110 5x EC50
2. 110 5x EC50 + LAM
3. 148 5x EC50
4. 148 5x EC50 + LAM
5. 152 5x EC50
6. 152 5x EC50 + LAM
7. 390 5x EC50
8. 390 5x EC50 + LAM
9. 514 5x EC50
10. 514 5x EC50 + LAM
11. 1% DMSO
12. 20 uM LAM

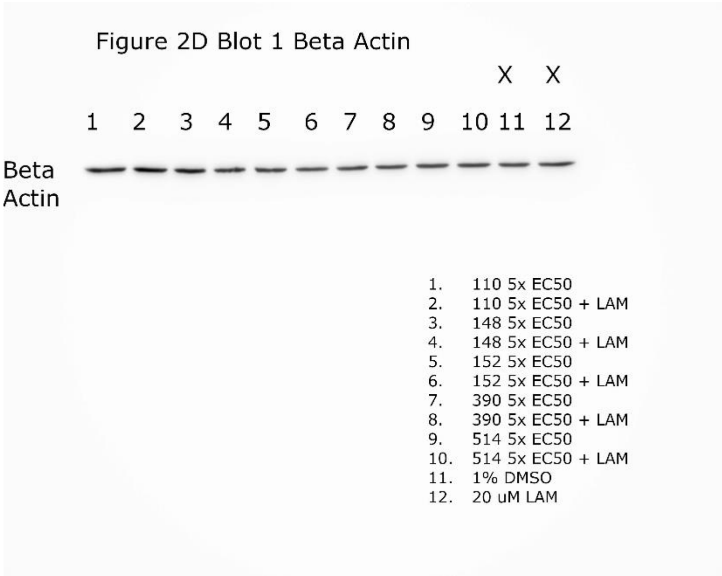
Figure 2D Blot 1 Beta Actin
X X
1 2 3 4 5 6 7 8 9 10 11 12
Beta Actin
1. 110 5x EC50
2. 110 5x EC50 + LAM
3. 148 5x EC50
4. 148 5x EC50 + LAM
5. 152 5x EC50
6. 152 5x EC50 + LAM
7. 390 5x EC50
8. 390 5x EC50 + LAM
9. 514 5x EC50
10. 514 5x EC50 + LAM
11. 1% DMSO
12. 20 uM LAM

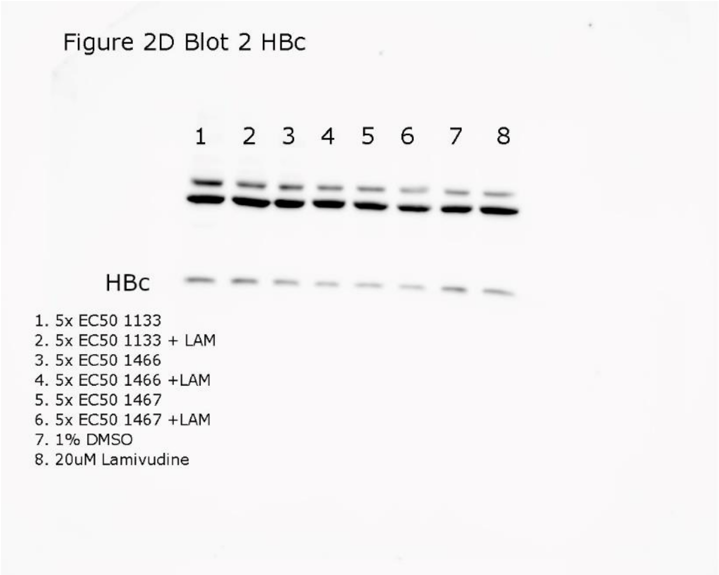
Figure 2D Blot 2 HBc
1 2 3 4 5 6 7 8
HBc
1. 5x EC50 1133
2. 5x EC50 1133 + LAM
3. 5x EC50 1466
4. 5x EC50 1466 +LAM
5. 5x EC50 1467
6. 5x EC50 1467 +LAM
7. 1% DMSO
8. 20uM Lamivudine

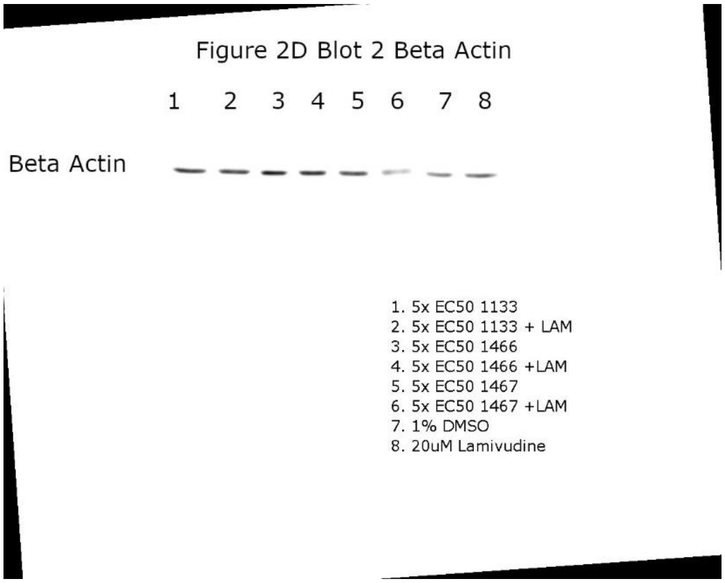
Figure 2D Blot 2 Beta Actin
1 2 3 4 5 6 7 8
Beta Actin
1. 5x EC50 1133
2. 5x EC50 1133 + LAM
3. 5x EC50 1466
4. 5x EC50 1466 +LAM
5. 5x EC50 1467
6. 5x EC50 1467 +LAM
7. 1% DMSO
8. 20uM Lamivudine

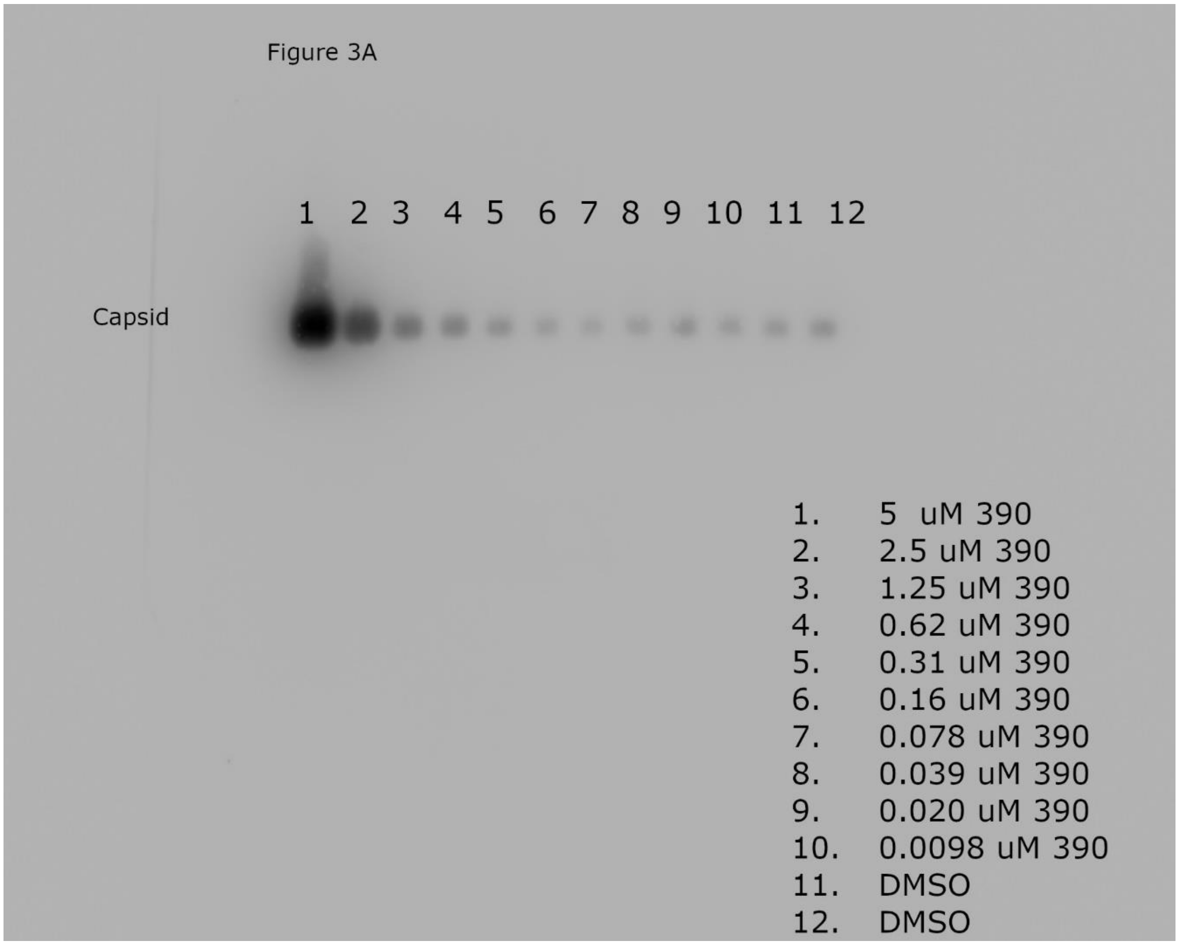
Figure 3A
1 2 3 4 5 6 7 8 9 10 11 12
Capsid
1. 5 uM 390
2. 2.5 uM 390
3. 1.25 uM 390
4. 0.62 uM 390
5. 0.31 uM 390
6. 0.16 uM 390
7. 0.078 uM 390
8. 0.039 uM 390
9. 0.020 uM 390
10. 0.0098 uM 390
11. DMSO
12. DMSO

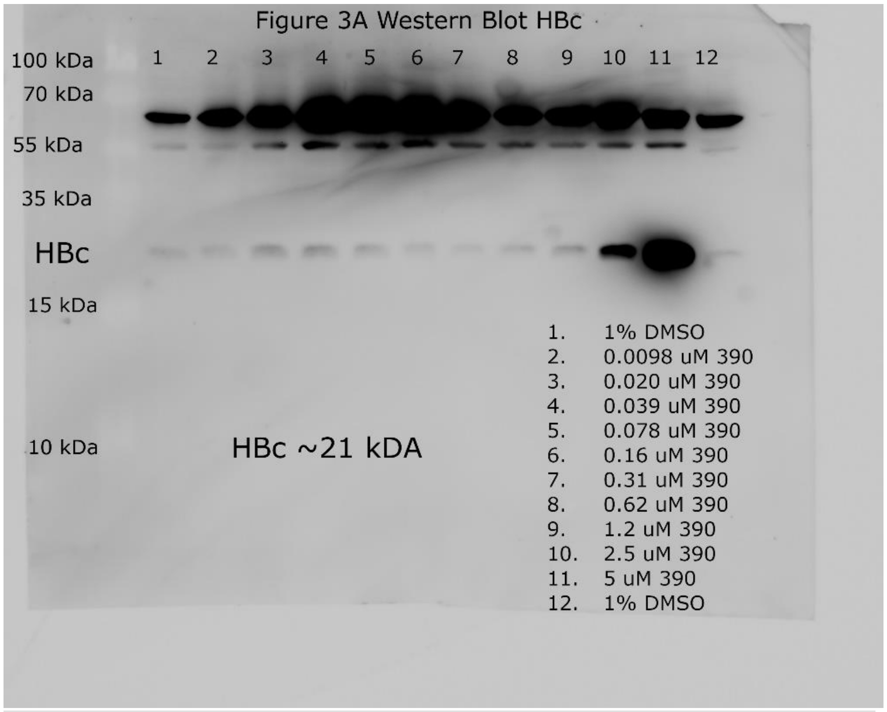
Figure 3A Western Blot HBc
100 kDa
70 kDa
55 kDa
35 kDa
HBc
15 kDa
10 kDa
1 2 3 4 5 6 7 8 9 10 11 12
HBc ~21 kDA
1. 1% DMSO
2. 0.0098 uM 390
3. 0.020 uM 390
4. 0.039 uM 390
5. 0.078 uM 390
6. 0.16 uM 390
7. 0.31 uM 390
8. 0.62 uM 390
9. 1.2 uM 390
10. 2.5 uM 390
11. 5 uM 390
12. 1% DMSO

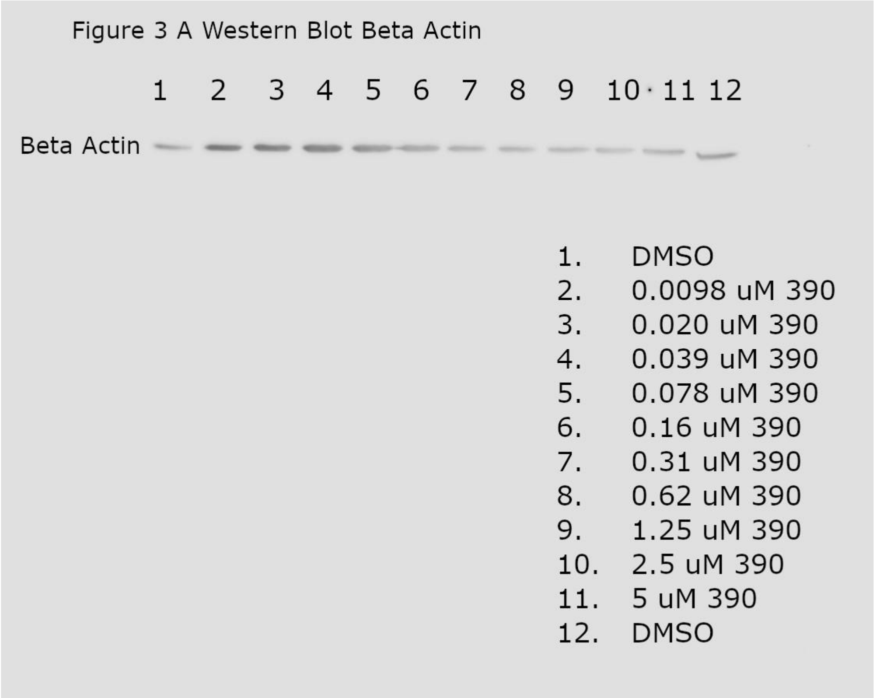
Figure 3 A Western Blot Beta Actin
1 2 3 4 5 6 7 8 9 10 11 12
Beta Actin
1. DMSO
2. 0.0098 uM 390
3. 0.020 uM 390
4. 0.039 uM 390
5. 0.078 uM 390
6. 0.16 uM 390
7. 0.31 uM 390
8. 0.62 uM 390
9. 1.25 uM 390
10. 2.5 uM 390
11. 5 uM 390
12. DMSO

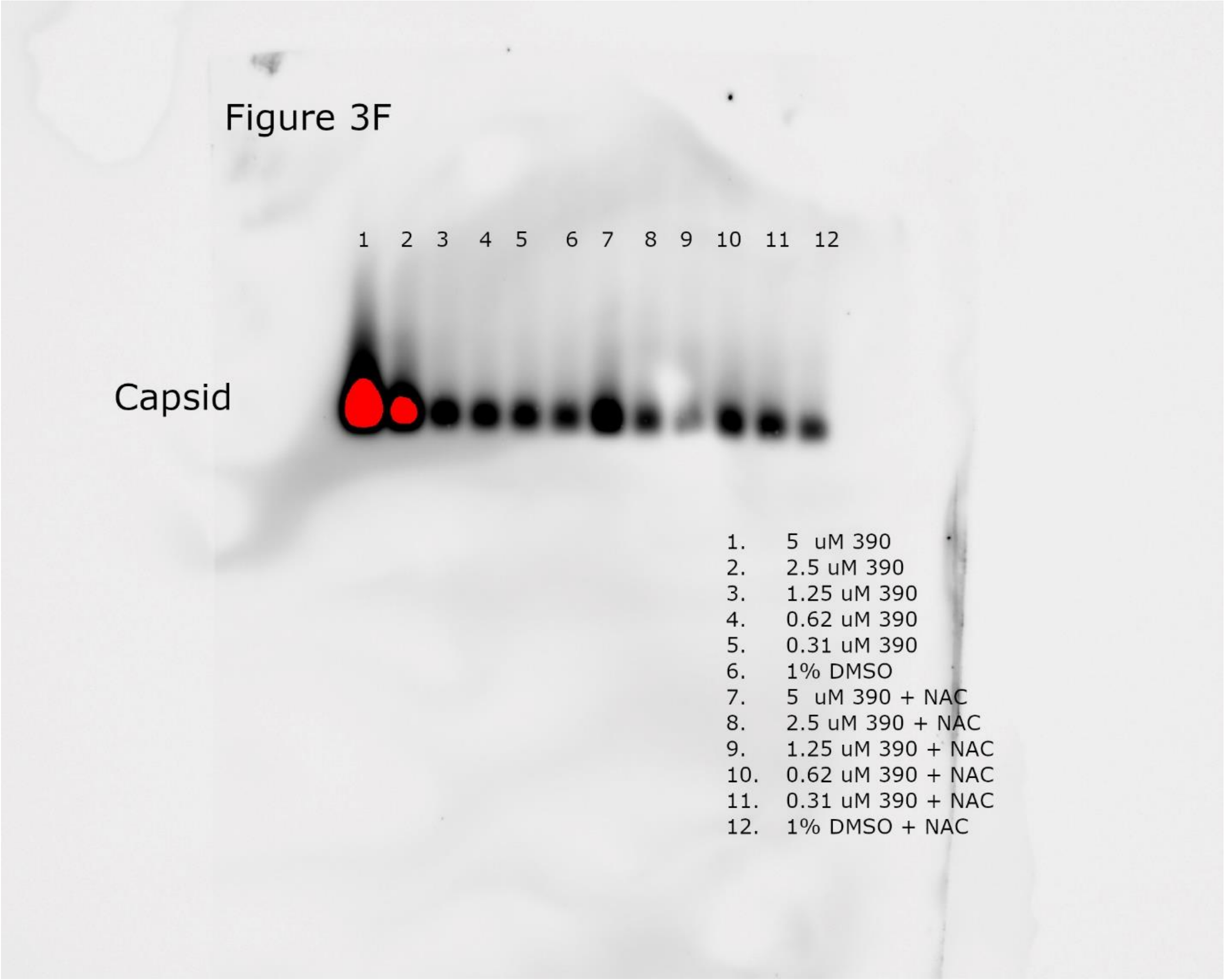
Figure 3F
1 2 3 4 5 6 7 8 9 10 11 12
Capsid
1. 5 uM 390
2. 2.5 uM 390
3. 1.25 uM 390
4. 0.62 uM 390
5. 0.31 uM 390
6. 1% DMSO
7. 5 uM 390 + NAC
8. 2.5 uM 390 + NAC
9. 1.25 uM 390 + NAC
10. 0.62 uM 390 + NAC
11. 0.31 uM 390 + NAC
12. 1% DMSO + NAC

Figure 4A

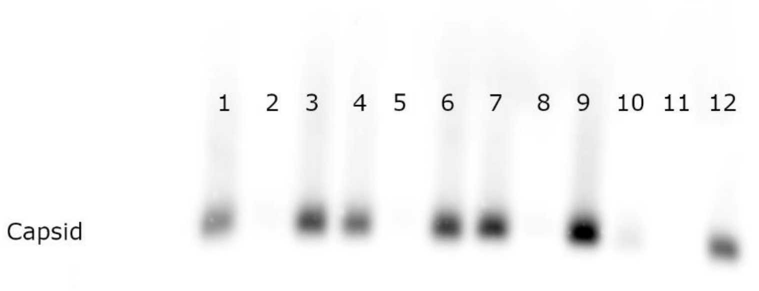

1. WT pCMV-HBV-LEII + 10uM 1466
2. WT pCMV-HBV-LEII + 1uM GLS4
3. WT pCMV-HBV-LEII + 1% DMSO
4. pCMV-HBV-LEII-D702A + 10uM 1466
5. pCMV-HBV-LEII-D702A + 1uM GLS4
6. pCMV-HBV-LEII-D702A + DMSO
7. pCMV-HBV-LEII-delta Bulge + 10uM 1466
8. pCMV-HBV-LEII-delta Bulge + 1uM GLS4
9. pCMV-HBV-LEII-delta Bulge + 1% DMSO
10. pCMV-HBV-LEII-YMHA + 10uM 1466
11. pCMV-HBV-LEII-YMHA + 1uM GLS4
12. pCMV-HBV-LEII-YMHA + 1% DMSO

Figure 4B

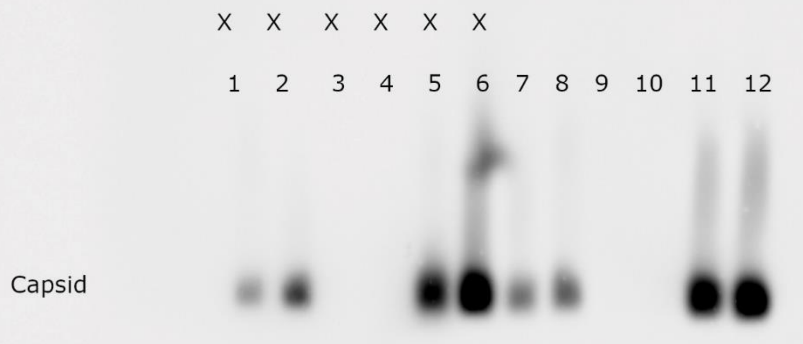

1. pCMV-HBc transfection 10uM 1466 Plate 1 well 1
2. pCMV-HBc transfection 10uM 1466 Plate 1 well 2
3. pCMV-HBc transfection 1uM GLS4 Plate 1 well 1
4. pCMV-HBc transfection 1uM GLS4 Plate 1 well 2
5. pCMV-HBc transfection 1% DMSO Plate 1 well 1
6. pCMV-HBc transfection 1% DMSO Plate 1 well 2
7. pCMV-HBc transfection 10uM 1466 Plate 2 well 1
8. pCMV-HBc transfection 10uM 1466 Plate 2 well 2
9. pCMV-HBc transfection 1uM GLS4 Plate 2 well 1
10. pCMV-HBc transfection 1uM GLS4 Plate 2 well 2
11. pCMV-HBc transfection 1% DMSO Plate 2 well 1
12. pCMV-HBc transfection 1% DMSO Plate 2 well 2

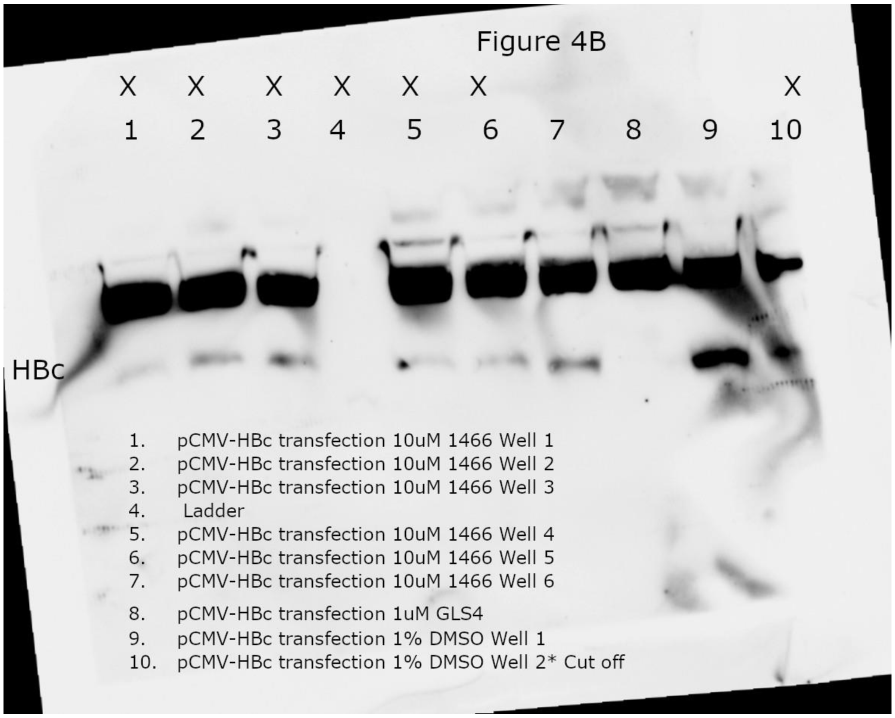
Figure 4B
X X X X X X X
1 2 3 4 5 6 7 8 9 10
HBc
1. pCMV-HBc transfection 10uM 1466 Well 1
2. pCMV-HBc transfection 10uM 1466 Well 2
3. pCMV-HBc transfection 10uM 1466 Well 3
4. Ladder
5. pCMV-HBc transfection 10uM 1466 Well 4
6. pCMV-HBc transfection 10uM 1466 Well 5
7. pCMV-HBc transfection 10uM 1466 Well 6
8. pCMV-HBc transfection 1uM GLS4
9. pCMV-HBc transfection 1% DMSO Well 1
10. pCMV-HBc transfection 1% DMSO Well 2* Cut off

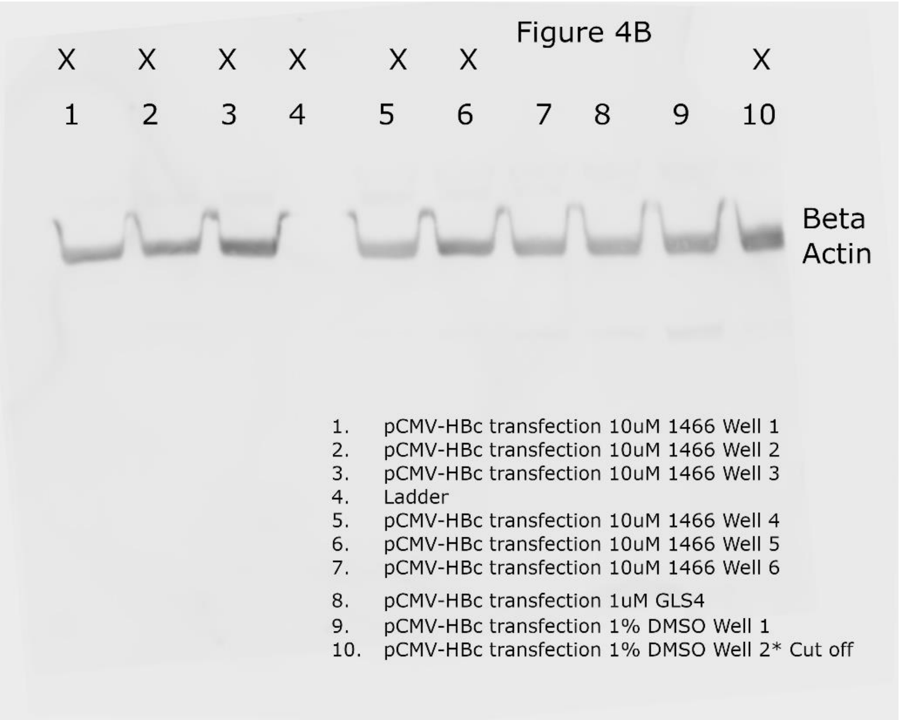
Figure 4B
X X X X X X X
1 2 3 4 5 6 7 8 9 10
Beta Actin
1. pCMV-HBc transfection 10uM 1466 Well 1
2. pCMV-HBc transfection 10uM 1466 Well 2
3. pCMV-HBc transfection 10uM 1466 Well 3
4. Ladder
5. pCMV-HBc transfection 10uM 1466 Well 4
6. pCMV-HBc transfection 10uM 1466 Well 5
7. pCMV-HBc transfection 10uM 1466 Well 6
8. pCMV-HBc transfection 1uM GLS4
9. pCMV-HBc transfection 1% DMSO Well 1
10. pCMV-HBc transfection 1% DMSO Well 2* Cut off

Figure 5B Capsid

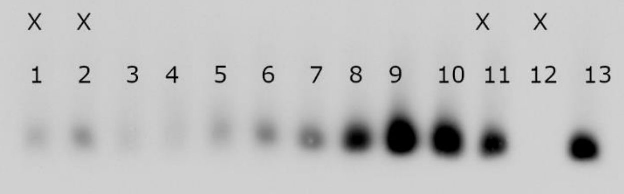

1. 20 uM 1466 n=1
2. 15 uM 1466 n=1
3. 10 uM 1466
4. 5 uM 1466
5. 2.5 uM 1466
6. 1.25 uM 1466
7. 0.625 uM 1466
8. 0.312 uM 1466
9. 0.156 uM 1466
10. 0.078 uM 1466
11. Lamivudine
12. Blank
13. Lamivudine

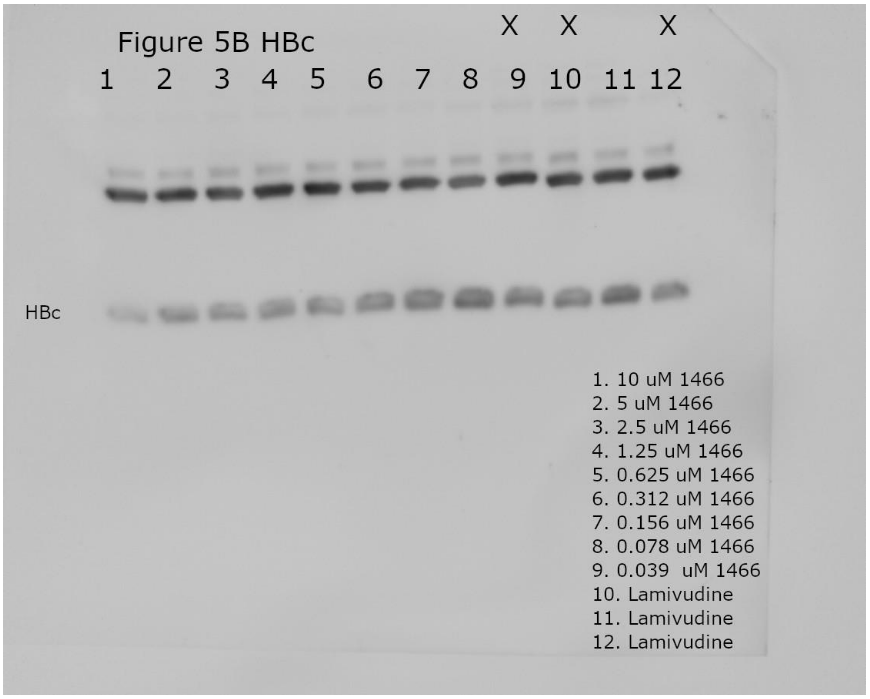

Figure 5 B Beta Actin

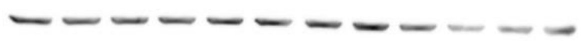

1. 10 uM 1466
2. 5 uM 1466
3. 2.5 uM 1466
4. 1.25 uM 1466
5. 0.625 uM 1466
6. 0.312 uM 1466
7. 0.156 uM 1466
8. 0.078 uM 1466
9. 0.039 uM 1466
10. Lamivudine
11. Lamivudine
12. Lamivudine

Figure 5C Capsid

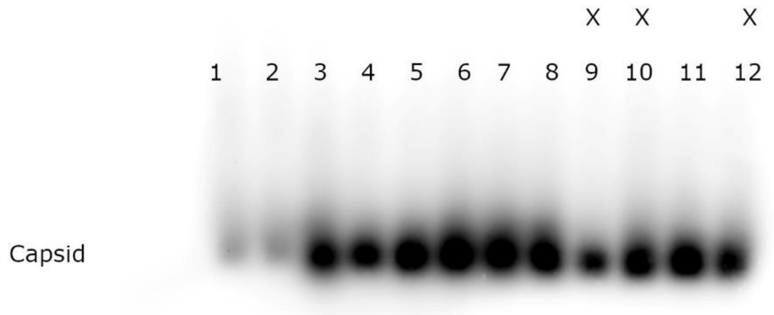

1. 10 uM 1466
2. 5 uM 1466
3. 2.5 uM 1466
4. 1.25 uM 1466
5. 0.625 uM 1466
6. 0.312 uM 1466
7. 0.156 uM 1466
8. 0.078 uM 1466
9. 0.039 uM 1466
10. Lamivudine
11. Lamivudine
12. Lamivudine

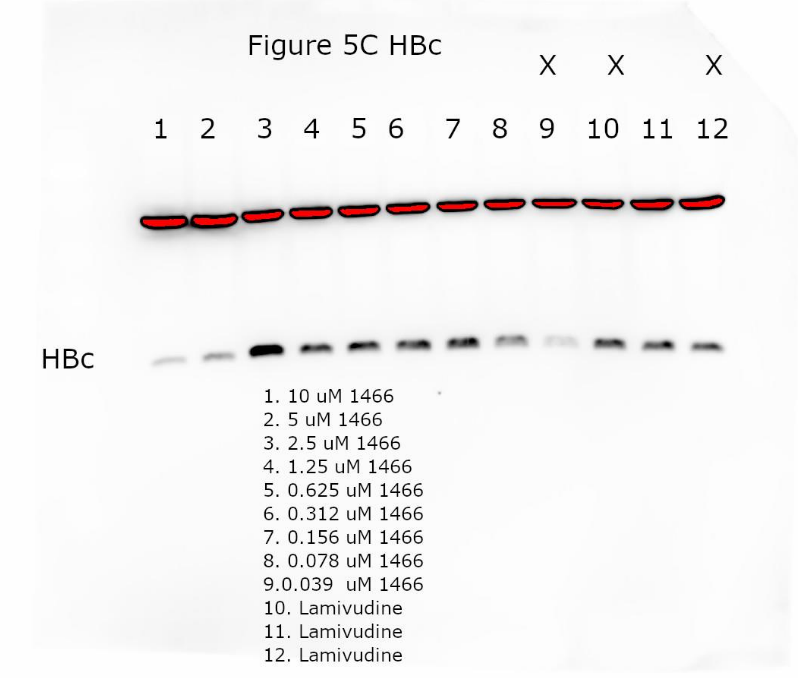
Figure 5C HBc
X X X
1 2 3 4 5 6 7 8 9 10 11 12
HBc
1. 10 uM 1466
2. 5 uM 1466
3. 2.5 uM 1466
4. 1.25 uM 1466
5. 0.625 uM 1466
6. 0.312 uM 1466
7. 0.156 uM 1466
8. 0.078 uM 1466
9.0.039 uM 1466
10. Lamivudine
11. Lamivudine
12. Lamivudine

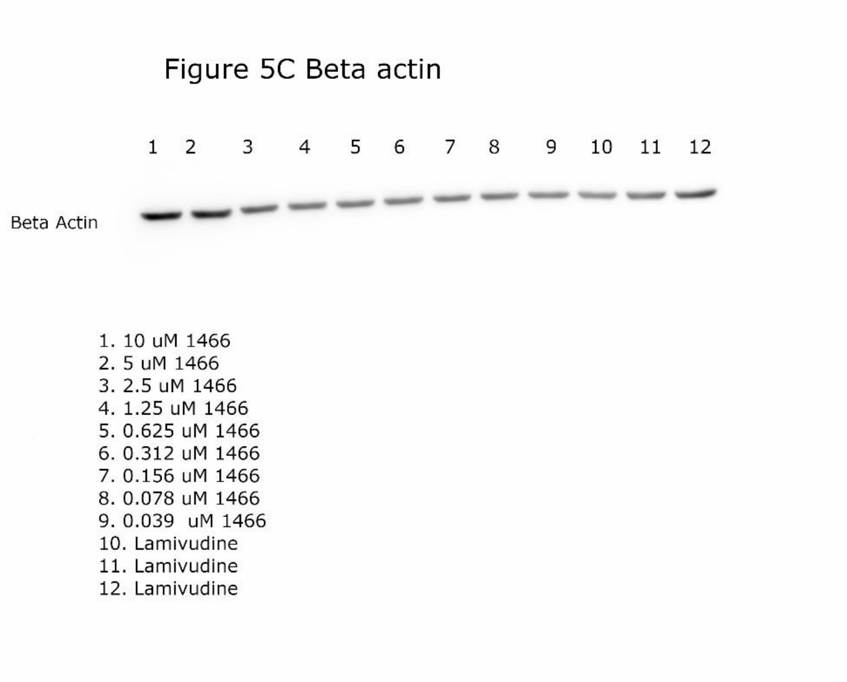
Figure 5C Beta actin
1 2 3 4 5 6 7 8 9 10 11 12
Beta Actin
1. 10 uM 1466
2. 5 uM 1466
3. 2.5 uM 1466
4. 1.25 uM 1466
5. 0.625 uM 1466
6. 0.312 uM 1466
7. 0.156 uM 1466
8. 0.078 uM 1466
9. 0.039 uM 1466
10. Lamivudine
11. Lamivudine
12. Lamivudine
