# Supplementary material for: Discovery of bimodal hepatitis B virus ribonuclease H and capsid assembly inhibitors
Source: PLoS Pathog. 2025 Feb 10;21(2):e1012920. doi: 10.1371/journal.ppat.1012920 (PMC11828405; doi:10.1371/journal.ppat.1012920)
Supplement: S1 Table — (DOCX) [file ppat.1012920.s008.docx]

^1^ GtD, Genotype D; GtA, Genotype A

**Table S1. Primer sets used to create mutant pCMV-HBV-LEII**

| Mutant | Sequence 5'-3' |
| --- | --- |
| Delta Bulge Forward | AAGCCTCCAAGCTGTGCC |
| Delta Bulge Reverse | TGGGACATGTACAAGAGATGATTAG |
| RNase H D702A Forward | GCAACCCCCACTGGCTGGGGCTTGGCC |
| RNase H D702A Reverse | GGCAGCAAACACTTGGCACAGACCAGGCC |
