# Supplementary material for: Discovery of bimodal hepatitis B virus ribonuclease H and capsid assembly inhibitors
Source: PLoS Pathog. 2025 Feb 10;21(2):e1012920. doi: 10.1371/journal.ppat.1012920 (PMC11828405; doi:10.1371/journal.ppat.1012920)
Supplement: S2 Table — (DOCX) [file ppat.1012920.s009.docx]

**Table S2. Primer and probe sets used in qPCR**

| Target ^1^ | Sequence 5'-3' |
| --- | --- |
| HBV GtD Core Forward | TGTATCGGGAAGCCTTAGAG |
| HBV GtD Core Probe | 56FAM/CCTCACCAT/ZEN/ACTGCACTCAGGCAA/3IABkFQ |
| HBV GtD Core Reverse | CCCAGGTAGCTAGAGTCATTAG |
| HBV GtA Core Forward | GACTTCCCT TCCGTCAGAG |
| HBV GtA Core Probe | 56FAM/ACCGCCTCA/ZEN/GCTCTGTATCGAGAA/3IABkFQ |
| HBV GtA Core Reverse | AGTATGGTGAGGTGAGCAATG |
| GAPDH Forward | GTGGTCTCCTCTGACTTCAAC |
| GAPDH Probe | 56FAM/TTGCCCTCA/ZEN/ACGACCACTTTGTCA/3IABkFQ |
| GAPDH Reverse | CCTGTTGCTGTAGCCAAATTC |

^1^ GtD, Genotype D; GtA, Genotype A
